# The 1831 CE mystery eruption identified as Zavaritskii caldera, Simushir Island (Kurils)

**DOI:** 10.1073/pnas.2416699122

**Published:** 2024-12-30

**Authors:** William Hutchison, Patrick Sugden, Andrea Burke, Peter Abbott, Vera V. Ponomareva, Oleg Dirksen, Maxim V. Portnyagin, Breanyn MacInnes, Joanne Bourgeois, Ben Fitzhugh, Magali Verkerk, Thomas J. Aubry, Samantha L. Engwell, Anders Svensson, Nathan J. Chellman, Joseph R. McConnell, Siwan Davies, Michael Sigl, Gill Plunkett

**Affiliations:** ^a^School of Earth and Environmental Sciences, University of St Andrews, St Andrews KY16 9TS, United Kingdom; ^b^Climate and Environmental Physics & Oeschger Centre for Climate Change Research, University of Bern, Bern 3012, Switzerland; ^c^Institute of Volcanology and Seismology, Russian Academy of Sciences, Petropavlovsk-Kamchatsky 683006, Russia; ^d^GEOMAR Helmholtz Centre for Ocean Research Kiel, Kiel 24148, Germany; ^e^Department of Geological Sciences, Central Washington University, Ellensburg, WA 98926; ^f^Department of Earth & Space Sciences, University of Washington, Seattle, WA 98195; ^g^Department of Anthropology, University of Washington, Seattle, WA 98195; ^h^Department of Earth and Environmental Sciences, University of Exeter, Penryn TR10 9EZ, United Kingdom; ^i^British Geological Survey, The Lyell Centre, Edinburgh EH14 4BA, United Kingdom; ^j^Centre for Ice and Climate, Section for the Physics of Ice, Climate, and Earth, Niels Bohr Institute, University of Copenhagen, Copenhagen 2200, Denmark; ^k^Division of Hydrologic Sciences, Desert Research Institute, Reno, NV 89512; ^l^Department of Geography, College of Science, Swansea University, Swansea, Wales SA2 8PP, United Kingdom; ^m^Archaeology & Palaeoecology, School of Natural and Built Environment, Queen’s University, Belfast BT9 3AZ, United Kingdom

**Keywords:** volcanoes, climate, ice cores, sulfur isotopes, tephra

## Abstract

Polar ice cores and historical records evidence a large-magnitude volcanic eruption in 1831 CE. This event was estimated to have injected ~13 Tg of sulfur (S) into the stratosphere which produced various atmospheric optical phenomena and led to Northern Hemisphere climate cooling of ~1 °C. The source of this volcanic event remains enigmatic, though one hypothesis has linked it to a modest phreatomagmatic eruption of Ferdinandea in the Strait of Sicily, which may have emitted additional S through magma–crust interactions with evaporite rocks. Here, we undertake a high-resolution multiproxy geochemical analysis of ice-core archives spanning the 1831 CE volcanic event. S isotopes confirm a major Northern Hemisphere stratospheric eruption but, importantly, rule out significant contributions from external evaporite S. In multiple ice cores, we identify cryptotephra layers of low K andesite-dacite glass shards occurring in summer 1831 CE and immediately prior to the stratospheric S fallout. This tephra matches the chemistry of the youngest Plinian eruption of Zavaritskii, a remote nested caldera on Simushir Island (Kurils). Radiocarbon ages confirm a recent (<300 y) eruption of Zavaritskii, and erupted volume estimates are consistent with a magnitude 5 to 6 event. The reconstructed radiative forcing of Zavaritskii (−2 ± 1 W m^−2^) is comparable to the 1991 CE Pinatubo eruption and can readily account for the climate cooling in 1831–1833 CE. These data provide compelling evidence that Zavaritskii was the source of the 1831 CE mystery eruption and solve a confounding case of multiple closely spaced observed and unobserved volcanic eruptions.

## Significance

One of the largest volcanic eruptions of the nineteenth century took place in 1831 CE. Although this event led to significant Northern Hemisphere climate cooling, the source of this eruption remains a mystery. Using evidence from well-dated ice cores and stratigraphic records we pinpoint Zavaritskii caldera, an extremely remote volcano located in the Kuril Islands (between Japan and Kamchatka), as the source of this eruption. By reconstructing its magnitude and radiative forcing we show that Zavaritskii can account for the climate cooling in 1831–1833 CE. These data provide a compelling candidate for this large-magnitude mystery eruption and demonstrate the climate-changing potential of these remote yet highly significant Kuril Island volcanoes.

Large-magnitude explosive volcanic eruptions inject sulfur dioxide (SO_2_) directly into the stratosphere, where it forms sulfate aerosols that reflect solar radiation and lead to significant global cooling (1, 2). For volcanologists, paleoclimatologists, and historians a particularly fascinating period is the final phase of the Little Ice Age, 1800–1850 CE, which is the coldest period in the last 500 y and is marked by a cluster of major volcanic events [identified by sulfate peaks in polar ice cores (3)]. These events include the 1815 CE eruption of Tambora in Indonesia, the 1835 CE eruption of Cosegüina in Nicaragua, and two unidentified eruptions in 1808/9 and 1831 CE. Although model simulations suggest these events played a significant role in global cooling (4), major uncertainties remain about the mass and injection height of sulfur and, crucially, the source of the mystery eruptions (5).

The 1808/9 and 1831 CE eruptions are the most recent large-magnitude volcanic stratospheric S injections that have yet to be matched to a known eruption source (6). Although much attention has been paid to the 1808/9 CE mystery eruption which injected ~19 Tg S into the stratosphere (5, 6), the 1831 CE eruption is also significant with a stratospheric injection of ~13 Tg S [larger than the ~7 to 10 Tg S calculated for the 1991 CE Pinatubo eruption (7)]. The 1831 CE eruption has been linked to climate cooling of 0.5 to 1 °C (*SI Appendix*, Fig. S1) and coincides with decreased rainfall in the African and Indian monsoon regions (4). It also precedes major famines in India [i.e., the 1832–1833 CE Madras or Guntur famine which affected most of eastern India (8)] and Japan [i.e., the 1832–1838 CE Tenpō famine which was particularly devastating in the north-east of the country (9)], both of which resulted from poor weather conditions and crop failure. Also remarkable are the historically documented atmospheric observations of a blue, purple, and green sun made at various Northern Hemisphere locations in August 1831 CE (10). Such phenomena were observed after the 1883 CE Krakatau eruption (Indonesia) and are caused by scattering and adsorption of solar radiation in a dense volcanic aerosol plume (11).

The 1831 CE eruption was initially attributed to Babuyan Claro volcano in the Philippines (12); however, Garrison et al. (13) traced various historical sources and found no firm evidence for an eruption at this time. Another notable candidate has been Ferdinandea (also known as Campi Flegrei Mar Sicilia or Graham Island) which is located ~50 km south-west of Sicily and erupted in July–August 1831 CE. This was a modest phreatomagmatic eruption with an erupted volume of 0.06 to 0.1 km^3^, or magnitude of 3.5 to 4.0 [where magnitude = log_10_[erupted mass (kg)] – 7, ref. 14]. Interestingly, Garrison et al. (10) showed an apparent westward progression of “blue” sun observations which initiate in Europe, propagate toward North America, and match the timing of the Ferdinandea eruption. Whether all these phenomena are tied to the aerosol veil of Ferdinandea and the sulfate deposited in the ice cores remains uncertain, although it is notable that the 1831 CE atmospheric phenomena are relatively short-lived (limited to August 1831 CE), in contrast to large-magnitude stratospheric eruptions (e.g. Tambora and Pinatubo) which last several years (15, 16). The magnitude of the Ferdinandea eruption is also unusually small for a climate-changing eruption. Its erupted volume and S estimates from melt inclusions yield a maximum magmatic S emission of only 0.3 Tg (10). Thus, a key corollary of the Ferdinandea hypothesis is that significant additional S (>10 Tg) was released by magma–crust interactions with evaporite rocks.

To obtain additional information about historical volcanic emissions we can turn to polar ice-core records. New ice-core analyses and dating have generated well-synchronized, subannually resolved records of chemical and particle fallout from major volcanic eruptions over the last 2500 y (3). Particle peaks can be investigated to identify cryptotephra horizons which can then be matched to proximal sources (17, 18). S isotopes of ice-core sulfate can be used to constrain plume injection height and source location since SO_2_ exposed to UV radiation in and above the stratospheric ozone layer acquires a unique S mass–independent fractionation [MIF (19, 20)]. As ice-core records provide precise constraints on eruption timings (21), linking unknown ice-core S peaks to a known volcanic source is important for reconstructing comprehensive regional and global volcanic records, improving volcanic forcing in climate modeling and understanding the societal impacts of large-magnitude eruptions. Here, we provide a much-needed reassessment of the ice-core record for the 1831 CE volcanic event, and through geochemical tephra correlation, we present a compelling candidate to explain this eruption conundrum.

## Results and Discussion

### Glaciochemical Records: Eruption Fallout and Timing.

**Fig. 1.**
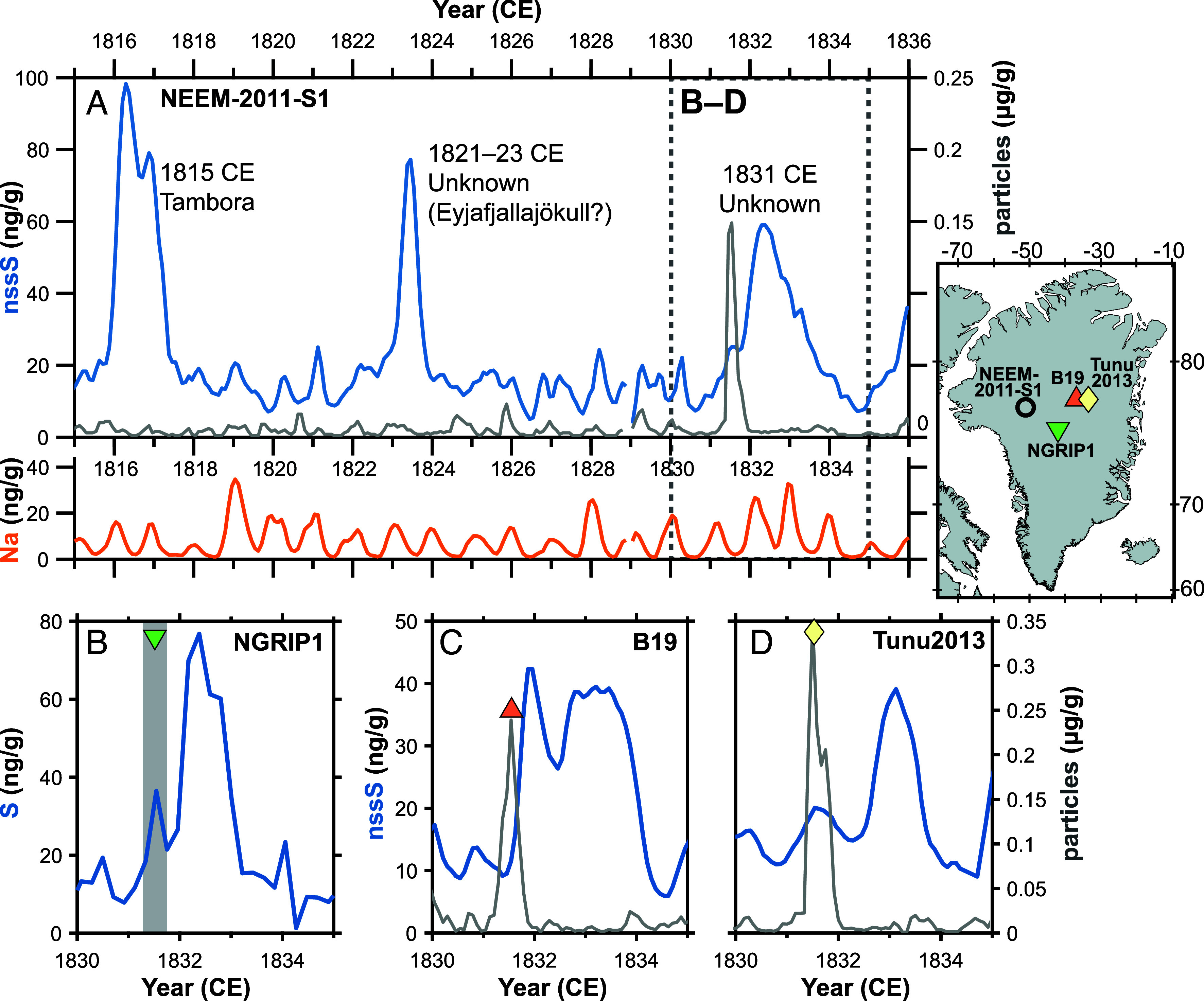
Glaciochemical records from Greenland ice-cores (NEEM-2011-S1 (*A*), NGRIP1 (*B*), B19 (*C*), and Tunu2013 (*D*)). Sulfur (S) and non-sea-salt sulfur (nssS) are shown on the left-hand axis (blue line; in ng/g). In the lower panel of (*A*) Na concentrations (orange line; in ng/g) from NEEM-2011-S1 are shown and reveal seasonal cycles with values peaking in midwinter due to increased storms (and hence increased transport of marine aerosol from the sea surface and sea ice). Particle concentrations (gray line; in μg/g) are shown on the right-hand axis and correspond to the 4.5 to 9.5 μm size fraction. Ice-core cryptotephra are shown by the colored symbols and are associated with the particle peaks. For NGRIP1 no particle concentration measurements were available but high-time-resolution subsampling allowed us to identify the precise depth interval of tephra (shaded gray). Note that there is a longer time offset between particle and S peaks at lower accumulation rate sites (B19 and Tunu2013, ~100 kg m^−2^ yr^−1^ of ice) compared to higher accumulation sites (NGRIP1 and NEEM, ~200 kg m^−2^ yr^−1^). Lower accumulation sites are more strongly affected by postdepositional processes (i.e. mixing, erosion, and redistribution of previous snow) and so the high accumulation sites (i.e. NGRIP1 and NEEM) best preserve the original stratigraphy.

Glaciochemical records from continuous flow analysis (NEEM-2011-S1, B19, and Tunu2013) and discrete samples (NGRIP1) are shown in Fig. 1. All cores show a major increase in S deposition spanning 1831–1834 CE, typically comprising an initial, and generally subsidiary S peak in 1831, followed by a larger peak in 1832–1833 CE. Another notable feature is the exceptional concentration of large (4.5 to 9.5 µm) insoluble particles that occurred prior to the main S peak. In Tunu2013 and NEEM-2011-S1 this particle peak is the largest in the 19th century, while in B19 it is the 3rd largest. Through optical and electron microscopy we confirmed that these particles are volcanic glass shards (with full geochemical results given in the following sections). The pattern of tephra deposition prior to peak S fallout is consistent with a mid-latitude volcanic emission. First, because ash particles fall out faster than sulfate aerosols [due to their larger size and mass (22, 23)], tephra from low-latitude eruptions are rarely transported and deposited in significant quantities to produce an obvious particle peak (c.f. 1815 Tambora, Fig. 1*A*). Second, for very proximal eruptions (e.g., from Iceland) particle and chemical fallout tend to be contemporaneous reflecting rapid transport mainly via tropospheric pathways (17, 24).

Seasonal glaciochemical cycles can further constrain the timing of the tephra fallout. Using the prominent 1815 CE Tambora signal in NEEM-2011-S1 as a fixed tie point, we counted seasonal Na cycles [which show a pronounced mid-winter peak due to increased storms and hence sea-salt flux (25)] and constrain the particle (tephra) fallout to summer 1831 CE (Fig. 1*A*). Sulfate fallout is sustained over at least two seasonal cycles (i.e., 2 y) and as this particle spike is identified in each ice core, we use it to synchronize the core chronologies.

A final observation is that the 1831 CE eruption is a bipolar event (i.e., there is synchronous S deposition in Antarctica and Greenland). However, a comparison of the relative magnitude of the peaks (*SI Appendix*, Fig. S2) reveals that S fallout in Greenland is ~6.5 times greater than in Antarctica (3, 26). A search of the Smithsonian Global Volcanism database (2024) shows only very minor (magnitude 2 to 3) eruptions in the Southern Hemisphere in 1831 CE. While we cannot rule out the possibility of an unidentified Southern Hemisphere eruption (27), the bipolar S peak with skewed deposition toward Greenland is consistent with a major mid-latitude Northern Hemisphere eruption.

### Sulfur Emission: Stratosphere–Troposphere Partitioning and Source.

**Fig. 2.**
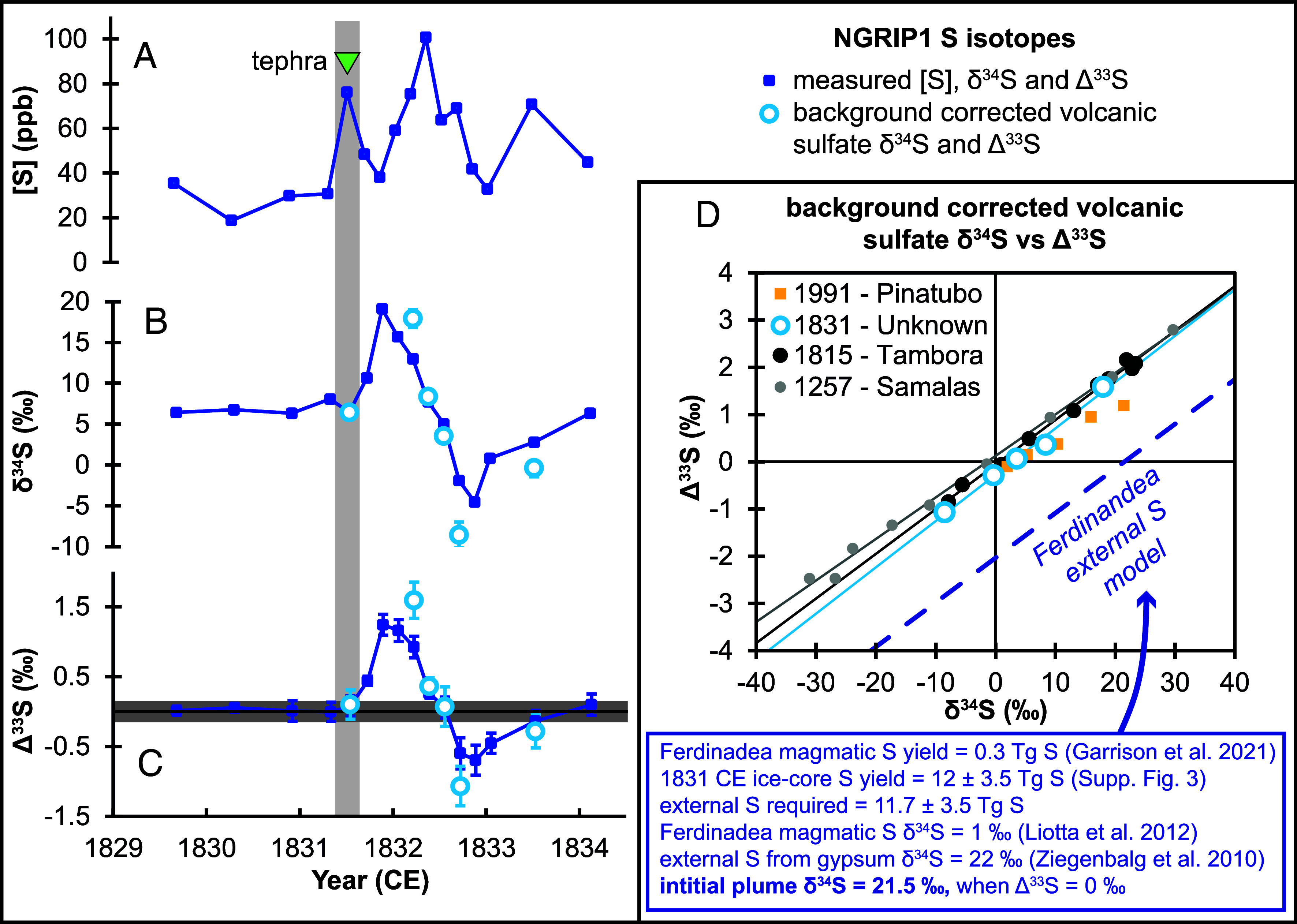
Sulfur concertation (*A*), δ^34^S (*B*), and Δ^33^S (*C*) time-series for NGRIP1. Blue-filled symbols are measured values, and the white-filled are background corrected values (for samples with >50 % volcanic sulfate (28, 29). In (*C*), the gray shaded area shows typical 2σ values of our non-MIF (0 ± 0.15 ‰) secondary standard [Switzer Falls river water (30)]. In *A*–*C*, the green triangle and gray shaded area show the depth interval of the subsample where cryptotephra were found. (*D*) shows background corrected volcanic sulfate values from large-magnitude volcanic events [1815 CE Tambora and 1257 CE Samalas (20) and 1991 CE Pinatubo (28)] compared to 1831 CE (this study, the 5 points from the stratospheric peak). The dashed blue line shows an S isotope mass balance model assuming Ferdinandea was the source of the 1831 CE ice-core S peak. The assumptions are summarized in the blue box and article text, but in short, large volumes of external S from Messinian gypsum horizons are required for Ferdinandea to reach the S loading suggested by the ice-core records and cannot explain the measured δ^34^S–Δ^33^S array. Ferdinandea model input is based on values from Garrison et al. (10), Liotta et al. (31), and Ziegenbalg et al. (32).

High-time-resolution measurements of S concentration, δ^34^S and Δ^33^S in NGRIP1 subsamples are shown in Fig. 2 *A–C*. Samples from 1829–1830 CE show limited variation in δ^34^S (6.3 to 6.8 ‰) and Δ^33^S values of ~0 ‰ [typical of background values (20)]. Over the main volcanic peak, we see a large positive to negative δ^34^S and Δ^33^S evolution, with maximum and minimum Δ^33^S values of 1.6 and −1.1 ‰ in background-corrected samples (Fig. 2*C*). The anomalous Δ^33^S values imply SO_2_ oxidation in the stratosphere at or above the ozone layer. The positive to negative Δ^33^S evolution is also a common feature of stratospheric eruptions (20, 28, 29). Nonzero Δ^33^S signals are generated during oxidation of SO_2_ to sulfate (which has a timescale of weeks to months), and therefore the multiyear Δ^33^S anomalies require physical separation of different Δ^33^S aerosol pools (29). These pools have different stratospheric residence times, do not reequilibrate, and therefore preserve this unique time-evolving Δ^33^S fallout in the ice core (Fig. 2*C*).

Interestingly, the initial S peak shows a Δ^33^S of 0.1 ‰, which is analytically indistinguishable from 0 ‰ (based on the 2σ values of our non-MIF secondary standard). This demonstrates that the initial S was from a lower altitude plume in the troposphere or lowermost stratosphere below the ozone layer. Importantly, the sample that immediately follows (i.e., on the declining limb of the initial peak) does show a detectable Δ^33^S value (of 0.4 ‰). To explain this feature, we must either invoke two near-simultaneous eruptions, one tropospheric and one stratospheric, or a single eruption which generated both tropospheric and stratospheric plumes. While we cannot unambiguously discriminate between these scenarios, the latter is consistent with glaciochemical evidence for a major mid-latitude Northern Hemisphere eruption and would explain the time evolution from an initial (rapid) deposition of tropospheric S and tephra particles, followed by prolonged fallout of stratospheric S over the month-years following.

Using an extensive array of bipolar ice cores, Toohey and Sigl (6) estimated a volcanic stratospheric S injection for the 1831 CE eruption to be 13 ± 3.5 Tg (1σ) and our S isotope data permit a minor revision of this value to 12 ± 3.5 Tg (since the initial S peak shows non-MIF Δ^33^S values, *SI Appendix*, Fig. S3). The ice-core S yield is far greater than the magmatic S yield predicted for Ferdinandea [0.3 Tg S, based on eruptive volumes and degassing (10)]. If Ferdinandea were responsible for the 1831 CE ice-core S, then large quantities of external S must be added to account for the amplitude of the S signal in the ice cores. Garrison et al. (10) suggest that additional S could be liberated by magma interactions with sedimentary rocks (i.e. Messinian evaporites). Mediterranean evaporite δ^34^S values are significantly higher [22 ‰ (32)] than typical magmatic δ^34^S [~1‰ (31)] and our isotopes allow us to test whether a significant portion of the sulfate deposited in Greenland came from an evaporitic source.

Magmatic S emissions have an initial mantle-like Δ^33^S of 0 ‰ and a characteristic δ^34^S that reflects both their mantle source and redox (33). For eruptions with stratospheric plumes, UV photochemical reactions fractionate S into positive and negative Δ^33^S pools, which fall out over several years and are deposited on polar ice sheets (19). In Fig. 2*D* we plot ice-core δ^34^S-Δ^33^S for identified stratospheric eruptions (20, 28). These show a characteristic linear array, which reflects the fact that the same process fractionates both δ^34^S and Δ^33^S, and that the sum of positive and negative isotope pools must approximate the initial Δ^33^S-δ^34^S [due to mass balance (29)]. An important mass balance constraint is that the best approximation of the initial δ^34^S is given when ice-core Δ^33^S ≈ 0 ‰. For Tambora and Samalas, initial δ^34^S show typical magmatic values of ~1 and −1.5 ‰, respectively, and for Pinatubo, the initial δ^34^S is 3.4 ‰, similar to the petrological reconstructions of 3.5 ‰ (34).

If Ferdinandea was responsible for the 1831 CE ice-core S, and the majority of the S (98 %, 11.7 Tg S) was derived from an external Messinian gypsum [with typical δ^34^S of 22 ‰ (32)], this would generate a δ^34^S-Δ^33^S array with an initial δ^34^S of 21.5 ‰ (i.e., when Δ^33^S = 0 ‰, as shown by the blue dashed line in Fig. 2*D*). The background-corrected 1831 CE S isotope data do not mirror this trend and show an initial δ^34^S of 2.7 ‰, similar to other identified eruptions. Likewise, if the initial tropospheric peak (Fig. 2 *A* and *B*) were Ferdinandea this too would lead to a significant increase in δ^34^S above the 6 ‰ background values. This increase is not observed either, allowing us to rule out large contributions from external sedimentary S associated with the Ferdinandea eruption as the source of the 1831 CE ice-core S deposits.

### Cryptotephra: Chemistry and Sources.

**Fig. 3.**
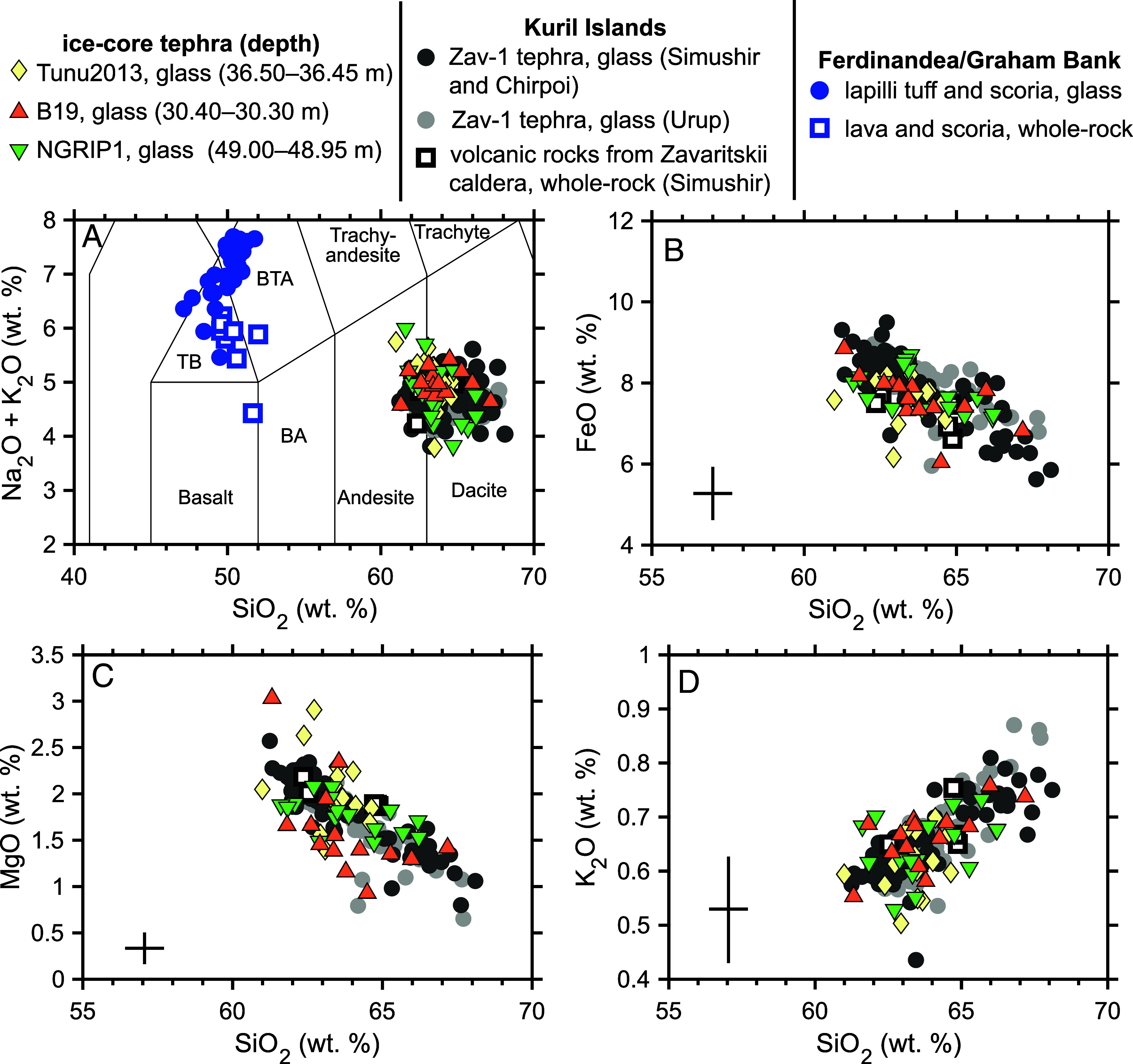
Major element geochemistry of the 1831 CE ice-core tephra compared to potential candidates. (*A*) is a total-alkali versus silica diagram and the abbreviations are TB: trachybasalt, BTA: basaltic-trachyandesite, and BA: basaltic-andesite. (*B*), (*C*), and (*D*) show major element biplots of SiO_2_ versus FeO, MgO and K_2_O, respectively. Triangles show ice-core tephra glass analyses, circles show glass analyses of proximal tephras and squares show whole-rock analyses of volcanic eruptives. Geochemical data for the 1831 CE eruption of Ferdinandea are shown in blue [this study and (35, 36)]. Zav-1 tephra originate from Zavaritskii caldera (Simushir Island, Kurils). Zav-1 ash and pumice samples from Simushir and Chirpoi Island (Fig. 4*A*) are shown by the large dark gray symbols. Zav-1 tephra found on Urup Island, ~140 km south-west of Zavaritskii caldera, are shown by large light gray circles. All Zav-1 tephra analyses are from this study (though the analyses were conducted at various times between 2009 and 2024), as detailed in Dataset S3. We also show additional geochemical measurements of volcanic rocks from Zavaritskii [after (37–39)]. Error bars give the maximum uncertainty in our ice-core tephra analyses (based on the 2σ values of the closest matrix-matched secondary standards).

**Fig. 4.**
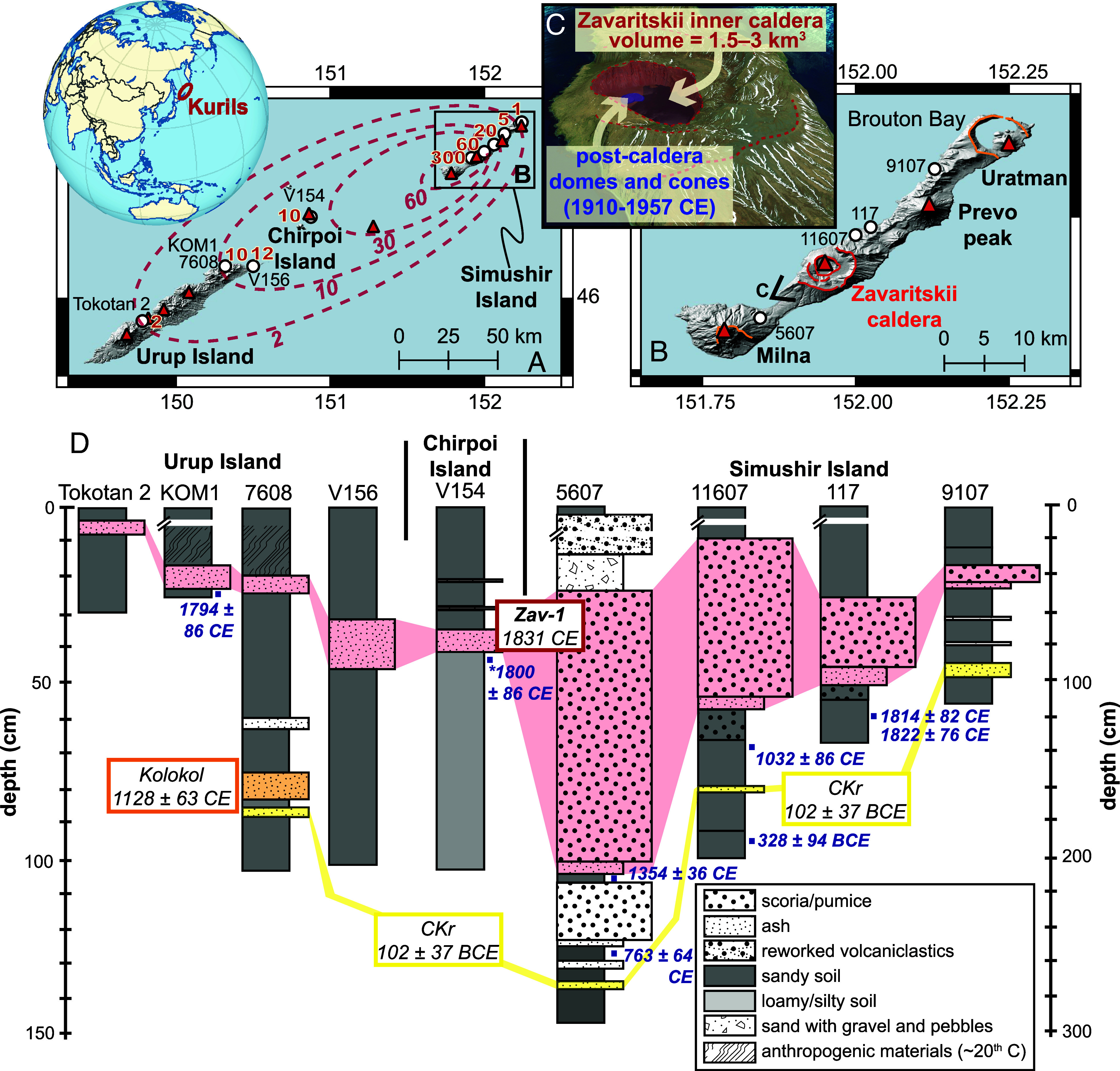
Location map of Zavaritskii caldera, Simushir Island, Kurils. (*A*) Volcanoes of Simushir and Urup Islands (red triangles) and sites where the Zav-1 tephra has been identified (white circles) with thicknesses in cm (red text). Approximate tephra isopachs are shown by the dashed red lines. (*B*) Detail of Simushir Island showing volcanoes, caldera outlines (in orange and red), and sampling locations labeled. (*C*) 3D view of the nested calderas of Zavaritskii, showing the youngest inner caldera (red) and the postcaldera lava domes (blue). (*D*) Stratigraphic columns showing the youngest volcaniclastic deposits on Simushir, Chirpoi Urup Island. The Zav-1 tephra, which is geochemically matched between these sites (Fig. 3) is shaded red. Ages for the Kolokol and CKr tephra layers are from Razjigaeva et al. (40) and Bergal-Kuvikas et al. (41), respectively. Anthropogenic materials found in the north of Urup Island include ~20th-century objects, i.e., tin cans and shoe leather. Calibrated radiocarbon ages (blue squares and text) are shown in years BCE/CE (mean ± 1σ). On Chirpoi Island the stratigraphy is from site V154 on Peschanaya Bay while the radiocarbon age (*) comes from a hearth deposit beneath Zav-1 also in Peschanaya Bay (42).

Large quantities of glass tephra shards coincide with the 1831 CE particle peaks in Tunu2013, B19, and NGRIP1 (Fig. 1 and *SI Appendix*, Fig. S5). These glass shards are 10 to 20 µm in size, and geochemical analyses indicate a single andesitic-dacitic population with characteristic low K (Fig. 3*A*). The ice-core tephra glass composition does not match the chemistry of proximal materials from the 1831 CE Ferdinandea eruption (Fig. 3*A*), and a comparison with regional geochemical datasets (*SI Appendix*, Fig. S4) shows greatest affinity to tephra from Japan and the Kuril Islands (Fig. 4).

Nineteenth century Japanese eruptions are generally well recorded, and as no eruptions are reported in 1831 CE (43), this led us to explore recent tephra deposits from the Kurils. The most compelling geochemical match for the ice-core tephra is a gray pumice fall deposit, the so-called “Zav-1” tephra, which is the youngest mappable volcaniclastic deposit identified on Simushir Island (44). It is found across the island and shows greatest thicknesses toward Zavaritskii caldera (Fig. 4 *A* and *B* and *SI Appendix*, Fig. S6). Our analysis of this proximal tephra glass shows that it matches the ice-core tephra on all major elements (Fig. 3). Volcanic rocks from Zavaritskii (45) also show excellent agreement with the ice-core tephra (Fig. 3). Moreover, on Chirpoi and Urup islands (~100 and ~140 km south-west of Simushir Island, respectively), the youngest tephra horizon also matches this characteristic low K andesitic-dacitic chemistry (40). A notable feature of volcanic glass of the Zav-1 tephra is the coherent geochemical trend between 61 and 68 % SiO_2_ (Fig. 3), which does not correlate with the abundance of microlites in glass shards. Similar trends in tephra chemistry have been observed for other Kuril Island eruptions (44) and in several Greenland ice-core tephra horizons which have been linked to major caldera-forming events (21) and indicate eruption of a chemically zoned magmatic system. In summary, the strong correlation between the Zav-1 tephra and the ice-core cryptotephra suggests that a caldera-forming eruption of Zavaritskii was the source of the tephra associated with the 1831 CE eruption.

### Zavaritskii Caldera: The Source of the Great 1831 CE Mystery Eruption.

Given the compelling geochemical match we now consider whether the timing and magnitude of the Zav-1 eruption are consistent with ice-core observations. Twelve radiocarbon dates (from soil and charcoal) both beneath and within Zav-1 are given in *SI Appendix*, Table S1. Obtaining precise dates for these samples is challenging because of their young age and a plateau in the radiocarbon calibration curve between 1700 and 1950 CE (which leads to large uncertainties in calibrated dates). Nevertheless, calibrated radiocarbon ages are mainly within the range of 1500–1900 CE. One of the strongest lines of evidence comes from Peschanaya Bay on Chirpoi Island where Zav-1 [the youngest mappable tephra layer, ≥10 cm thick, Fig. 4*D* (44)] overlies cultural materials (a rusted gun and muscovite imported for windowpanes) known to be from the Russian colonial period [1700s to early 1800s (42)]. Thus, there is strong evidence to support the Zav-1 eruption occurring within the last ~300 y and its link to the 1831 CE ice-core tephra.

To place constraints on the tephra fallout volume we use the available tephra thickness measurements (Fig. 4 and *SI Appendix*, Fig. S6). Isopachs show thicker tephra deposits toward the south-west, and we used these to calculate the bulk volume of tephra fall using three commonly used models [the exponential model (46), the power law model (47), and the Weibull model (48)]. These models (*SI Appendix*, Fig. S7) yield a bulk deposit volume of 3.3 to 4.5 km^3^. Assuming a deposit density of 900 kg m^−3^ we calculate a magnitude of 5.5. This is comparable to the climactic dacitic pumice-fall deposit (layer C and the submarine ash layer) of the 1991 Pinatubo eruption which has a magnitude of 5.8 (49, 50), and at least an order of magnitude lower than the Plinian ash fall of the 1815 CE Tambora eruption (magnitude 7.0, ref. 51.

The total mass of erupted deposits can also be approximated by the caldera volume (51). Zavaritskii comprises a set of nested calderas (Fig. 4*C*) and based on geomorphology, the inner most caldera is the youngest (i.e., caldera walls are steepest, and vegetation is limited). Postcaldera volcanism began around 1910 CE (37) and was marked by small scoria cone and lava dome eruptions. This supports caldera formation prior to the 1900s; consistent with radiocarbon dates of Zav-1. Assuming the caldera was created by the withdrawal of magma from the 1831 CE eruption, the caldera volume would suggest an erupted dense rock equivalent (DRE) volume of 1.5 to 3 km^3^ (magnitude 5.6 to 5.8). As this calculation does not account for any pre-eruption topography and volcaniclastics that infilled the caldera, it represents a minimum estimate. The caldera estimate of erupted volume is somewhat larger than the fall deposit volume (which would equate to a DRE of 1.3 to 1.7 km^3^) and is explained by the fact that caldera volume accounts for both the Plinian fallout and ignimbrite phases.

Combining the eruptive volume constraints with estimated S concentrations in pre-eruptive melt inclusions and degassed matrix glass allows a first-order approximation of the S output using the equation:

$$E_{S}=\frac{M_{v}\left(1-W_{\mathrm{xls}}\right)\left(C_{\mathrm{inclusion}}-C_{\mathrm{matrix}}\right)}{100},$$

where E_S_ is the S emission in kg, M_V_ is the mass of erupted magma in kg, W_xls_ is the mass fraction of crystals in the magma and C_inclusion_ − C_matrix_ is the difference between the average S concentrations of the inclusions and the matrix glass in wt. % (52). For Zav-1, Mv is 7.4–8.1 × 10^13^ kg (using the tephra thickness and caldera volume estimates) and W_xls_ is 0.1 to 0.2 (based on petrological imaging, *SI Appendix*, Fig. S5). Melt inclusion investigation of Zav-1 eruptive materials has yet to be undertaken, although analyses of other Holocene eruptive units of Zavaritskii have been reported previously (53). Assuming Zav-1 melt inclusions are comparable to past eruptions then pre-eruptive S (C_inclusion_) is ~900 ppm (the maximum content of orthopyroxene hosted melt inclusions) and matrix glass S (C_matrix_) is ~150 ppm (measured on Zav-1 tephra from Chirpoi island, Dataset S3). This calculation estimates an output of 2–6 Tg S for Zav-1 and given the tephra isopach and caldera volume represent minimum estimates, this S yield is also likely to represent a minimum. Our petrological S yield is significantly larger than the magmatic S output known for Ferdinandea [0.3 Tg (10)] but lower than, though within error, of the ice-core volcanic stratospheric injection estimate of 12 ± 7 Tg S (2σ) (*SI Appendix*, Fig. S3). Discrepancies between petrological and ice-core S yields are well known and reflect uncertainties on eruptive volume, the presence of a separate fluid phase in the magma prior to eruption, and/or the atmospheric pathway and processing of the plume between its source and the ice sheet (54). Nevertheless, our age constraints, erupted volume, and S loading estimates all support a major eruption from Zavaritskii caldera in the 1700–1900 CE period.

Our evidence establishes Zavaritskii as the prime candidate for the 1831 CE mystery eruption and raises several key questions. First, could such a large eruption have gone unrecorded? Japanese records mention various atmospheric phenomena apparently occurring in 1831 CE, including dry fog, abnormal color of sun and moon, Bishops ring, and volcanic hair [volcanic ash] falling from sky (55). These observations occurred prior to the Tenpō famines (1832–1838 CE) and lend support to Zavaritskii as a foreign (yet relatively proximal) volcanic source (although they warrant further investigation). Detailed historical records from the Kurils are extremely limited and there is little information on the occupation of Simushir Island during the 18th and 19th centuries. Formal historical accounts (56) suggest that the island was occupied sporadically since the 1760s by small villages of Ainu (Indigenous people of northern Japan and the Kurils), as well as small colonies of Russian settlers and conscripted Aleuts (Indigenous people of the Aleutian Islands) as part of the Russian–American (fur trading) Company. The main area of settlement was in the very north of the island at Brouton (Broughton) Bay (Fig. 4*B*). It is unclear whether there was any permanent settlement here in 1831 CE, but it is likely that the population would have been few [even in the 1870s when a small village existed in Brouton Bay the population numbered only ~50 people (56)]. Given the Zav-1 eruption took place ~30 km away, and that tephra isopachs (Fig. 4*B*) show limited ash fallout over Brouton Bay, it seems plausible that such an event could have gone unrecorded.

A second question is: Can the Zav-1 eruption account for the climate cooling observed in 1831–1833 CE (*SI Appendix*, Fig. S1)? To test this, we reconstructed its radiative forcing (RF) using the volcanic aerosol model EVA_H (57) with the forcing efficiency scaling of Marshall et al. (58). A detailed description of the model and setup are provided in the Methods, but for Zav-1 we input the latitude of Zavaritskii and a summer injection of 12 ± 7 Tg S (2σ, 9) at a height of 23 ± 12 km above sea level (the median value for SO_2_ injection for magnitude 5 to 6 eruptions, *SI Appendix*, Figs. S8 and 9). Using this approach, we calculate a peak global monthly mean stratospheric aerosol optical depth (SAOD) of 0.11 ± 0.08 and a peak global monthly mean effective RF of −2 ± 1 W m^−2^ (*SI Appendix*, Figs. S10 and 11, all uncertainties expressed as 95% CI). These values are roughly half that of Tambora but comparable to the 1835 CE Cosegüina eruption and the 1991 CE eruption of Pinatubo (−2 to −3 W m^−2^, refs. 59–61). Given that the low latitude (15.1 °N) eruption of Pinatubo led to Northern Hemisphere cooling of 0.5 to 0.6 °C (62), it is reasonable to conclude that Zav-1, an eruption of similar magnitude but which concentrated aerosols in the Northern Hemisphere (*SI Appendix*, Fig. S11), produced a comparable if not slightly amplified Northern Hemisphere temperature response (0.5 to 1 °C) in agreement with climate records (*SI Appendix*, Fig. S1).

A final question is: Do the westward propagating “blue sun” phenomena in 1831 CE relate to the eruption of Zavaritskii or Ferdinandea?, A key feature of the 1831 CE phenomena is their coincidence in space and time with the Ferdinandea eruption, and their short duration [limited to August 1831 CE (10)]. These observations are in stark contrast to those reported after large-magnitude stratospheric eruptions [e.g. Tambora (16) and Pinatubo (15)] where atmospheric phenomena (anomalously colored sunrises and sunsets, and dark lunar eclipses) are reported globally over several years. Although our S isotope data (Fig. 2*D*) rule out Ferdinandea as the source of the ice-core S peak, the extremely short-lived nature of the August 1831 CE blue sun phenomena and their predominance in the Mediterranean and eastern North America, are more consistent with the smaller-magnitude Ferdinandea eruption. An interesting parallel can be drawn with volcanic events in 44 BCE when a relatively minor eruption of Etna produced a series of unusual atmospheric phenomena across the Mediterranean while a far larger caldera-forming eruption of Okmok (Alaska) led to Northern Hemisphere climate cooling (23). 1831 CE appears to be a similar case of closely timed eruptions, with the more significant climate-changing event, Zav-1, going undetected until now.

Our identification of Zavaritskii as the source of the 1831 CE ice-core S peak improves the global inventory of large, climate-impacting volcanic events and enhances the regional volcanic record by yielding a precisely dated tephra isochron for the Kuril Islands. Moreover, the new constraints on the location, S mass, and injection height of the 1831 CE eruption will be of significant use to the modeling community, allowing improved estimates of RF and regional climate impacts of this eruption and, more generally, a better understanding of climate sensitivity to large-magnitude, mid-latitude Northern Hemisphere eruptions. Future work on the Zav-1 eruption should focus on isopleth (clast size) mapping to better estimate the plume height, and melt inclusion analysis to better constrain the volatile budget; this would permit more accurate ash dispersion and S injection modeling. Further research is also needed into the 1832–1833 CE Madras [India (8)] and 1832–1838 CE Tenpō famines [Japan (9)] which closely follow the 1831 CE eruption. While climate records (*SI Appendix*, Fig. S1) and reconstructions (4) do support a decrease in Northern Hemisphere temperatures and monsoon rainfall at this time, a thorough examination of historical records is vital to understanding both the regional climate impacts and the sociopolitical factors which may have governed the societal response to this significant volcano-climate forcing.

## Conclusions

Our study reveals that a large-magnitude eruption of Zavaritskii caldera (Simushir Island, Kurils) occurred in summer 1831 CE. Although a modest eruption of Ferdinandea also occurred in summer 1831 CE, and might be responsible for various aerosol optical phenomena, our ice-core evidence demonstrates that ash, as well as the tropospheric and stratospheric S deposited in Greenland derive from Zavaritskii. Radiocarbon and archaeological evidence from proximal locations corroborate the eruption timing, while volume estimates confirm an eruption of magnitude 5 to 6, sufficient to explain the ice-core-based stratospheric S loading of 12 ± 3.5 Tg. The reconstructed peak global mean RF of −2 ± 1 W m^−2^ of Zavaritskii is comparable to other magnitude 5 to 6 eruptions (i.e. 1991 CE Pinatubo and 1835 CE Cosegüina) and is in line with the 0.5 to 1 °C cooling observed in tree ring and instrumental temperature records. Our finding opens up a wide array of future research to better understand the dynamics of 1831 CE Zav-1 eruption and its wider climatic and societal impacts. More broadly, it underscores the importance of constraining eruption style, timing, and magnitude of these remote but hugely significant Kuril volcanoes.

## Methods

### Glaciochemistry.

High-time-resolution chemical, elemental, and particle concentration records were determined for the NEEM-2011-S1, Tunu2013, and B19 ice cores using the unique continuous ice core analytical system at the Ultra Trace Chemistry Laboratory at the Desert Research Institute (DRI). This system is detailed by Sigl et al. (3) and McConnell et al. (63) and includes two High Resolution Inductively Coupled Plasma Mass Spectrometers (HR-ICP-MS) for elemental measurements (e.g., S), as well as an Abakus laser-based insoluble particle detector. The latter registers insoluble particle concentrations in 2.6 to 4.5 µm and 4.5 to 9 µm size fractions and was used to guide subsampling for cryptotephra.

### Sulfur Isotopes.

For NGRIP1 we subsampled the 1831–1834 CE S peak at 4 to 5 cm resolution (yielding a nominal 2 to 3-mo time resolution). These samples were analyzed for S isotopes (^34^S, ^33^S, and ^32^S) and cryptotephra. We achieved this by centrifuging the sample and removing all but the bottom 2 to 3 mL of supernatant for isotope analysis and leaving the remainder for cryptotephra sampling. For S isotopes we followed the column chemistry protocol of Burke et al. (20) though adapted this using an automated approach with a Prepfast-MC. For this a single PFA column, with 2 PTFE frits and 50 μL of AG1-X8 resin was used for all samples. To regenerate the resin for each sample, the resin was washed with 600 μL 1.1 M HCl, 1,000 μL MilliQ water, 600 μL 1.6 M HNO_3_, another 600 μL 1.1 M HCl, and 600 μL 0.06 M HCl. Samples were loaded with 100 μL 0.01% (v/v) distilled HCl, plus an additional 9 to 180 μL of additional MilliQ water to counteract the effects of evaporation in the autosampler chamber. The column was then matrix washed with 750 μl MilliQ water, before the sulfate fraction was collected in 550 μL 0.5M HNO_3_. Following column chemistry all samples were dried down and redissolved such that they formed solutions of 40 μmol Na_2_SO_4_ in 0.5 M HNO_3_. This was to match the sample matrix to our in-house bracketing standard (20). In-house secondary standards [Switzer Falls river water (30)], and procedural blanks were prepared and analyzed in an identical manner to the unknowns (and their δ^34^S and Δ^33^S were consistent with previous measurements; see Dataset S2).

We also analyzed large (10 to 20 cm, ~7 to 10 mo) background samples taken in the years before and after the 1831–1834 CE S peak. These samples have low S concentrations (20 to 35 ppb) and Δ^33^S of ~0 ‰ and were used to remove the background S contributions (mainly from marine sources) and determine endmember isotope values of the volcanic emissions. To do this we follow the equation of refs. 28 and 29:

[1] $$\delta_{\mathrm{volc}}=(\delta_{\mathrm{meas}}-f_{\mathrm{bkgd}}\delta_{\mathrm{bkgd}})/f_{\mathrm{volc}},$$

where $\delta_{\mathrm{volc}}$, $\delta_{\mathrm{meas}}$, and $\delta_{\mathrm{bkgd}}$ are the δ^34^S or δ^33^S of the volcanic, measured, and background sulfate values, respectively. $f_{\mathrm{bkgd}}$ is the mass fraction of the total sulfate in the background ($f_{\mathrm{bkgd}}={\left[{\mathrm{SO}}_{4}\right]}_{\mathrm{bkgd}}/{\left[{\mathrm{SO}}_{4}\right]}_{\mathrm{sample}}$), and $f_{\mathrm{volc}}$ is the mass fraction of volcanic sulfate ($f_{\mathrm{volc}}=1-f_{\mathrm{bkgd}}$). In our plots, we only consider $\delta_{\mathrm{volc}}$ for samples with greater than 50 % volcanic sulfate ($f_{\mathrm{volc}}>0.5$).

### Volcanic Sulfate Deposition.

Since the S isotope results show that the Greenland ice-core S deposit for 1831–1833 CE comprises both tropospheric (Δ^33^S ≈ 0 ‰) and stratospheric (Δ^33^S ≠0 ‰) sulfate (Fig. 2), it is necessary to update the current volcanic stratospheric S injection [VSSI, (6)] which assumes that all S deposited in this period is stratospheric in origin. To do this we calculated the volcanic sulfate deposition using S and sulfate concentration records from three Greenland ice cores with high snow accumulation (i.e., 19 to 41 cm per year): Summit2010 (64), D4 (65), and NGRIP1 (66). For NGRIP1, we use the volcanic deposition as calculated by ref. 66. For the monthly resolved D4 and Summit2010 ice cores, we use a similar approach as described in detail in ref. 67. This calculation estimates the median annual cycle of monthly resolved background sulfate deposition over the time period 1741–1870 CE for Summit2010 [1733–1875 CE for D4 (67)] after exclusion of all monthly values influenced by major volcanic eruptions (e.g., following Tambora 1815 CE). This annual background cycle is subtracted from the total monthly resolved sulfate depositions to isolate the volcanic sulfate depositions at these ice-core sites. The initial tropospheric peak extends from 1831.25–1831.75 CE and represents 5 to 14 % of the total cumulative deposition over 1831–1833 CE (*SI Appendix*, Fig. S3). Using the average value of these three cores (8 %) allows us to revise the VSSI estimate (6) from 13 ± 3.5 Tg to 12 ± 3.5 Tg S (1σ).

### Cryptotephra.

For cryptotephra subsamples, we mounted all insoluble particles on microprobe slides (Tunu2013) or stubs (B19 and NGRIP1) using epoxy, and then polished using diamond paste (6, 3, 1 µm) and aluminum oxide slurry (0.25 µm). Major and minor element geochemical analysis of ice-core cryptotephra was performed by electron microprobe analysis (EPMA) at the University of Bern (B19), Queen’s University Belfast (Tunu2013), and the University of St Andrews (NGRIP1). Fresh glass fragments of proximal tephra from our candidate eruption were analyzed by EPMA. These tephra were sampled by previous field campaigns in the Kurils and were analyzed at the University of Washington, GEOMAR (Kiel), and St Andrews between 2009 and 2024 (Dataset S3). we provide a detailed description of the sampling, tephra stratigraphy, and analyses of these samples. Secondary standards were analyzed concurrently and used to monitor EPMA accuracy and precision; full details of these values as well as the instruments and operating conditions for each session are given in Dataset S3. In the figures all data are normalized to 100 % on an anhydrous basis.

### Simulations of Volcanic Aerosol Optical Properties and RF.

To evaluate the RF of our candidate (Zav-1, Zavaritskii) eruption we used the Easy Volcanic Aerosol model [EVA, (68)]. The EVA reconstruction uses volcanic stratospheric S injection (VSSI) constraints from an array of bipolar ice-core records [i.e. eVolv2k (6)] and is the recommended volcanic forcing dataset for climate model simulations of Phase 4 of the Paleoclimate Model Intercomparison Project [PMIP, (69)]. The EVA model was recently updated by Aubry et al. (57) to include mass, latitude, and height of the injected SO_2_, and was further calibrated against a full set of satellite era observations (rather than Pinatubo alone). This extended model is used here and referred to as EVA_H (57). We use EVA_H to convert the ice-core VSSI into satellite aerosol optical depth (SAOD) at 550 nm. We then use the relationship of Marshall et al. (58) to estimate the global monthly mean effective RF from aerosol optical properties:

[2] $$RF=-20.7\times (1-e^{-\Delta SAOD}),$$

where RF is in Wm^−2^ and the scaling prefactor may vary between −23.1 and −17.3 Wm^−2^ depending on the eruption season and latitude. The ΔSAOD corresponds to the anomaly of SAOD at 550 nm with respect to the background level, i.e. the SAOD only due to volcanic aerosols. Due to the computationally inexpensive nature of our models, we propagate uncertainties on the eruptions and model parameters by generating two 1,000-member ensembles. The mass of SO_2_, injection height, EVA_H parameters, and SAOD-RF scaling factor are resampled within their uncertainties using Gaussian distributions. SO_2_ mass and latitude have the largest influence on SAOD, height also affects it with higher plume height leading to a greater lifetime and SAOD (70, 71). A large range of eruption parameters are considered (detailed below) and these uncertainties are reflected in our range of forcing estimates.

### SO_2_ Injection Source Parameters and Model Comparison.

For our 1831 CE reconstructions the latitude is 46.9 °N (i.e. the latitude of Zavaritskii). The date is set at 01/08/1831 (DD/MM/YYYY) based on the presence of the 1831 CE Zav-1 tephra particle spike between two seasonal Na peaks (indicative of winter/spring storms, Fig. 1*A*). The stratospheric S mass is 12 ± 7 Tg (2σ) which represents VSSI for 1831 CE (6) with the contribution from the initial tropospheric S peak removed (*SI Appendix*, Fig. S3).

Accurate constraints on plume height rely on isopleth mapping using maximum pumice or lithic diameter. These data are unavailable for Zav-1 and so to estimate the plume height we used two different approaches. In the first approach, we compiled maximum column height distributions for magnitude 4 to 7 eruptions and dacitic compositions from the Large-Magnitude Explosive Volcanic Eruptions (LaMEVE) database (72) (*SI Appendix*, Fig. S8). The 1831 CE eruption of Zavaritskii (Zav-1) is a magnitude 5 to 6 (see main text) and so using the median plume heights from LaMEVE this gives a range of 30 to 36 km. In the second approach we used the maximum column height versus magnitude relationship (*SI Appendix*, Fig. S9) from a more carefully curated dataset of tephra fall deposits from Eychenne and Engwell (73). We fit their data with an exponential function:

[3] $$\ln_{plumeheightabovevent\left(km\right)}=0.61457+0.52554\times \mathrm{magnitude},$$

and used the magnitude estimated for the 1831 CE Zav-1 tephra (5.5; see main text) to estimate a plume height of 33 km above vent level (a.v.l.). We note there is good agreement between the two approaches and opt for a maximum plume height range of 30 to 36 km. It is important to note that these plume heights mostly represent maximum isopleth-derived top heights. It is well known that isopleth-based height represents an upper bound on the top height of the plume and that the main peak of SO_2_ injection occurs at lower altitudes. Aubry, Engwell et al. (74) examined this relationship for well-observed volcanic events and showed that the average ratio of the isopleth height to the mean top height was 1.45. They also showed that the ratio of SO_2_ injection heights to top heights was 0.97, and so using these values we convert our isopleth-derived maximum plume height estimate (30 to 36 km a.v.l.) to an SO_2_ injection range of 20 to 24 km a.v.l. (using a scaling factor of 1.49). Given the topography of Zavaritskii caldera and the vent heights of neighboring volcanoes on Simushir Island (Fig. 4*B*), it is likely that the eruptive vent of Zav-1 was ~0.5 to 1.5 km above sea level. EVA_H requires SO_2_ injection as a height above sea level and so this gives a range of 20.5 to 25.5 km. While this is our best estimate for the SO_2_ injection height, there are large uncertainties and so in our EVA_H model we decided to take a conservative approach and set the height as 23 ± 12 km above sea level.

We refer to the simulation ensemble described above, using our SO_2_ injection parameters and the EVA_H model, as ***Zavaritskii-EVA_H***. For reference we compare this to the volcanic SO_2_ injection source parameters currently used in the eVolv2k inventory (6). This ensemble, referred to as ***eVolv2k-EVA_H***, has a stratospheric mass of 13 ± 7 Tg S and is attributed to Babuyan Claro (Philippines) with latitude of 19.5°N, default date of 01/01/1831. To constrain injection height (H) we use the default mass of SO_2_ (M), following the relationship used in the Coupled Model Intercomparison Project (CMIP7) historical dataset:

[4] $$H={\mathrm{aM}}^{b},$$

where a = 15.61 and b = 0.1585. We then sample the height within an uncertainty of σ = 0.33 × H.

## Supplementary Material

Appendix 01 (PDF)

Dataset S01 (XLSX)

Dataset S02 (XLSX)

Dataset S03 (XLSX)

## Data Availability

All study data are included in the article and/or supporting information.
