# Supplementary material for: The 1831 CE mystery eruption identified as Zavaritskii caldera, Simushir Island (Kurils)
Source: Proc Natl Acad Sci U S A. 2024 Dec 30;122(1):e2416699122. doi: 10.1073/pnas.2416699122 (PMC11725861; doi:10.1073/pnas.2416699122)
Supplement: Supplementary file 1 — Appendix 01 (PDF) [file pnas.2416699122.sapp.pdf]

Supporting Information for
The 1831 CE mystery eruption identified as Zavaritskii caldera, Simushir Island (Kurils)

William Hutchison*[1], Patrick Sugden[1], Andrea Burke[1], Peter Abbott[2], Vera V. Ponomareva[3], Oleg Dirksen[3], Maxim V. Portnyagin[4], Breanyn MacInnes[5], Joanne Bourgeois[6], Ben Fitzhugh[7], Magali Verkerk[8], Thomas J. Aubry[8], Samantha L. Engwell[9], Anders Svensson[10], Nathan J. Chellman[11], Joseph R. McConnell[11], Siwan Davies[12], Michael Sigl[2] and Gill Plunkett[13]

[1]School of Earth and Environmental Sciences, University of St Andrews, St Andrews, UK
[2]Climate and Environmental Physics & Oeschger Centre for Climate Change Research, University of Bern, Bern, Switzerland
[3]Institute of Volcanology and Seismology, Russian Academy of Sciences, Piip Boulevard, 9, 683006, Petropavlovsk-Kamchatsky, Russia
[4]GEOMAR Helmholtz Centre for Ocean Research Kiel, 24148 Kiel, Germany
[5]Department of Geological Sciences, Central Washington University, 400 E. University Way, Ellensburg, 98926, WA, USA
[6]Department of Earth & Space Sciences, University of Washington, Seattle, WA, USA
[7]Department of Anthropology, University of Washington, Seattle, WA, USA
[8]Department of Earth and Environmental Sciences, University of Exeter, Penryn, UK
[9]British Geological Survey, The Lyell Centre, Edinburgh, UK
[10]Centre for Ice and Climate, Section for the Physics of Ice, Climate, and Earth, Niels Bohr Institute, University of Copenhagen, Copenhagen, 2200, Denmark
[11]Division of Hydrologic Sciences, Desert Research Institute, Reno, Nevada, USA
[12]Department of Geography, College of Science, Swansea University, Swansea, Wales, UK
[13]Archaeology & Palaeoecology, School of Natural and Built Environment, Queen's University Belfast, Belfast, UK

William Hutchison
Email: wh39@st-andrews.ac.uk

This PDF file includes:

Supporting Information Text
Figures S1 to S11
Table S1
SI References

Other supporting materials for this manuscript include the following:

Datasets S1 to S3

Supporting Information Text

Zav-1 tephra stratigraphy

The volcanic stratigraphy of the Central Kuril Islands has been investigated by numerous authors over the last few decades. Much of this research was an international collaborative effort between US, Russian and Japanese scientists as part of the International Kuril Island Project (IKIP) in 2000 and the Kuril Biocomplexity Project (KBP) between 2006–2010. The Zav-1 tephra was formally named and identified by Nakagawa et al. (1) who mapped the layer across Simushir Island and also identified correlatives on Chirpoi and Urup islands. A type locality for Zav-1 is the west coast of Simushir Island at a site referred to as Nakatomari (07-SM-34, Figure S6). Here, Nakagawa et al. (1) identified Zav-1 as the youngest tephra deposit (it is 30 cm thick and is found beneath 15 cm of modern soil). Two samples of Zav-1 from 07-SM-34 were provided by these authors, analyzed using EPMA at the University of St Andrews (Dataset S3) and show an excellent match to the 1831 CE ice-core shards (Fig. 3).

Further stratigraphic sections and correlations in the Central Kuril Islands were made by tephra and radiocarbon dating during the KBP. A number of these sections (which include Zav-1) have been published in Razzhigaeva et al. (2), MacInnes et al. (3) and Fitzhugh et al. (4, 5), and all of them are available as part of the KBP archive (6). Relevant to our work are sites V154 (Fig. 4) and 2008-24 which are both located in Peschanaya Bay, Chirpoi Island. At these sites the youngest recognizable tephra horizon was sampled and analysed by EPMA at University of Washington (V154 in 2009) and GEOMAR (2008-24 in 2015), and we later re-analysed V154 by EPMA at the University of St Andrews (in 2024). All these analyses match proximal Zav-1 geochemistry. Also relevant are sites V156, KOM1 and Tokotan 2 located on Urup Island. We note that in some KBP reports KOM1 is referred to as Novo 1 – 2006, while Tokotan 2 is sometimes labelled Shabalinka rechka. EPMA of V156 tephra was initially conducted at University of Washington in 2009, and in 2024 we analyzed the youngest tephra layer at all these sites (i.e. V156, KOM1 and Tokotan 2) at the University of St Andrews (detailed in Dataset S3). These tephras all show good geochemical overlap with Zav-1.

Additional fieldwork was conducted on north Urup Island under the framework of the KBP and was reported by Razjigaeva et al. (7). These authors report various stratigraphic sections, tephra and radiocarbon age correlations that link their youngest tephra (i.e. 7508, Fig. 4) to the Zav-1 layer of Nakagawa et al. (1). Finally, field work was also conducted on Simushir Island in June 2011 by O. Dirksen who sampled the youngest tephra layers from the western coast of Zavaritskii caldera (sites 01101 and 01118, Figure S6). EPMA of these tephra was conducted at Moscow State University in 2011. These data also show a clear match to Zav-1 geochemistry and are also included in Dataset S3.

Supplementary Figures

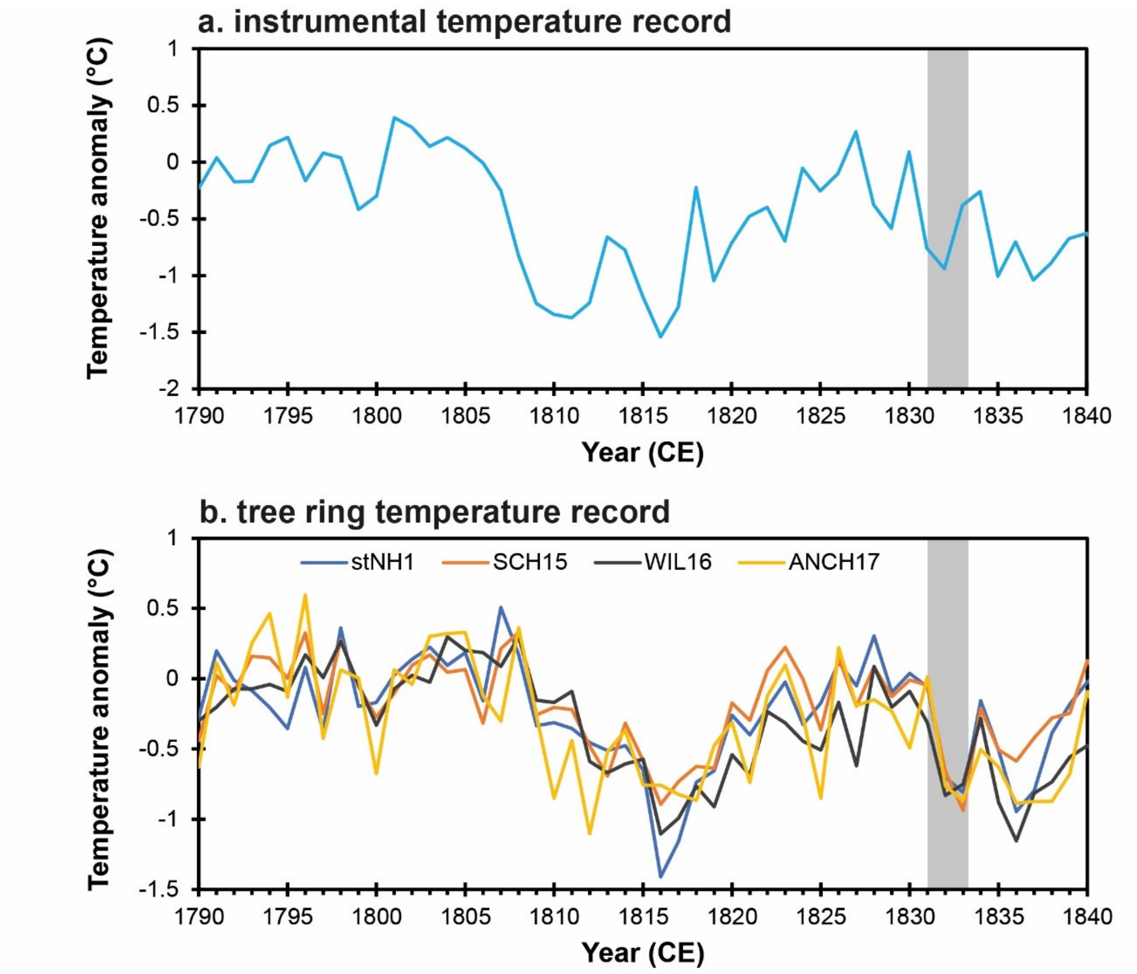

Figure S1: Time series of instrumental (a) and tree ring (b) temperature anomalies spanning 1790–1840 CE. The period 1831–1833 CE is shaded grey. Instrumental temperature records are from Berkeley Earth [www.berkeleyearth.org, (8)] and show estimated global land average temperatures. These represent annual temperature anomalies relative to the 1790–1807 CE mean (a period without major volcanic eruptions). Tree ring temperature reconstructions are from key Northern Hemisphere datasets: stNH1 (9), SCH15 (10), WIL16 (11) and ANCH17 (12). These report May-August (MJJA) temperature anomalies also relative to the 1790–1807 CE mean. We note that instrumental records show a declining temperature trend between 1827 and 1837 CE (i.e. prior to the 1831 and 1835 CE stratospheric S injections). This might be a reflection of Northern Hemisphere climate modes, and we note that reconstructions of both North Atlantic Oscillation (13) and the Atlantic Multidecadal Oscillation (14, 15) show negative phases at this time which might explain the overall pattern of 2–3 year volcanic cooling signals superimposed on a longer period of cooler temperatures.

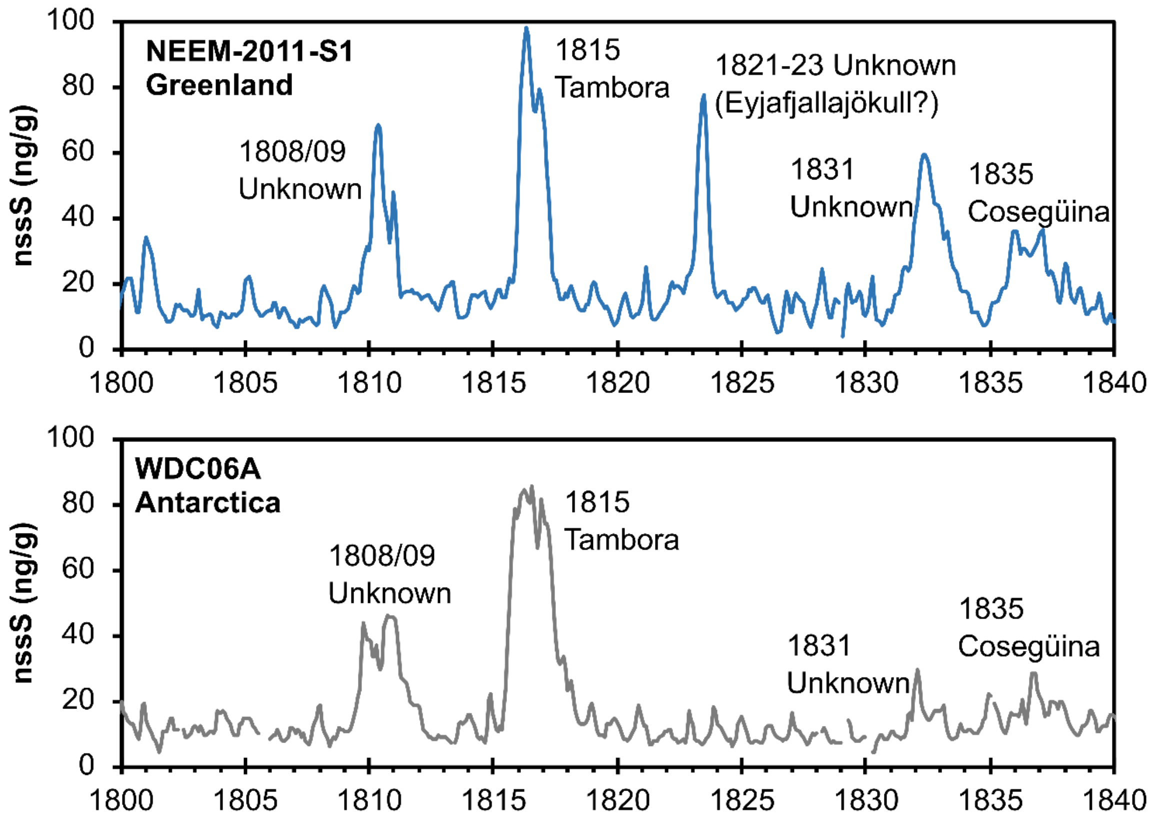

Figure S2: Time series of non-sea salt sulfur (nssS) for Greenland (NEEM-2011-S1) and Antarctica (WDC06A). Note the much greater magnitude of the 1831 CE in Greenland compared to Antarctica, indicative of a Northern Hemisphere eruption.

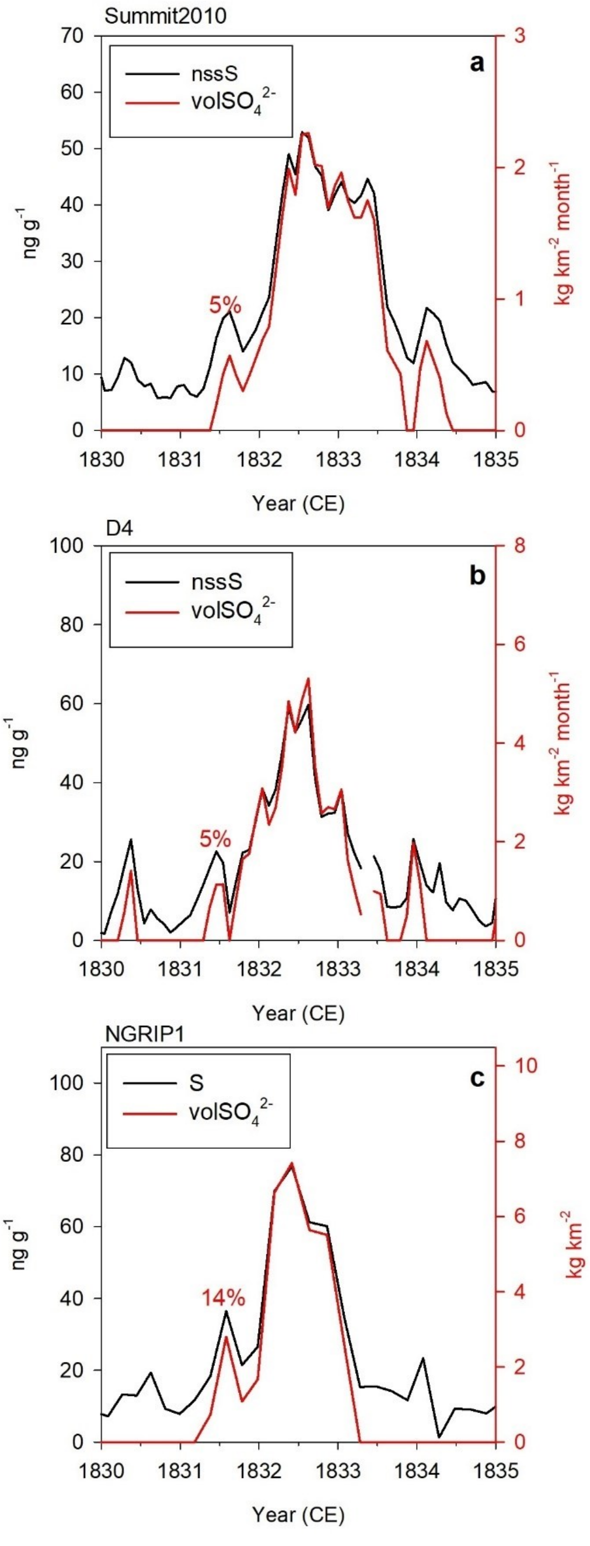

Figure S3: High-resolution sulfur (S) and non-sea salt sulfur (nssS) and volcanic sulfate deposition records from central Greenland ice cores: (a) Summit2010 (16), (b) D4 (17) and (c) NGRIP1 (18). The initial peak, 1831.25–1831.75 CE, is attributed to tropospheric sulfate (Fig. 2) and so we calculate the percentage of total cumulative S deposition (over 1831–1833 CE) that can be attributed to this (the remainder being stratospheric). This initial peak ranges from 5–14 % of the total volcanic sulfate deposition. Taking the average value (8 %) we can revise the previous volcanic stratospheric S injection

(VSSI) estimate of Toohey and Sigl (19), which assumed all sulfate was stratospheric, from 13 ± 3.5 Tg to 12 ± 3.5 Tg S.

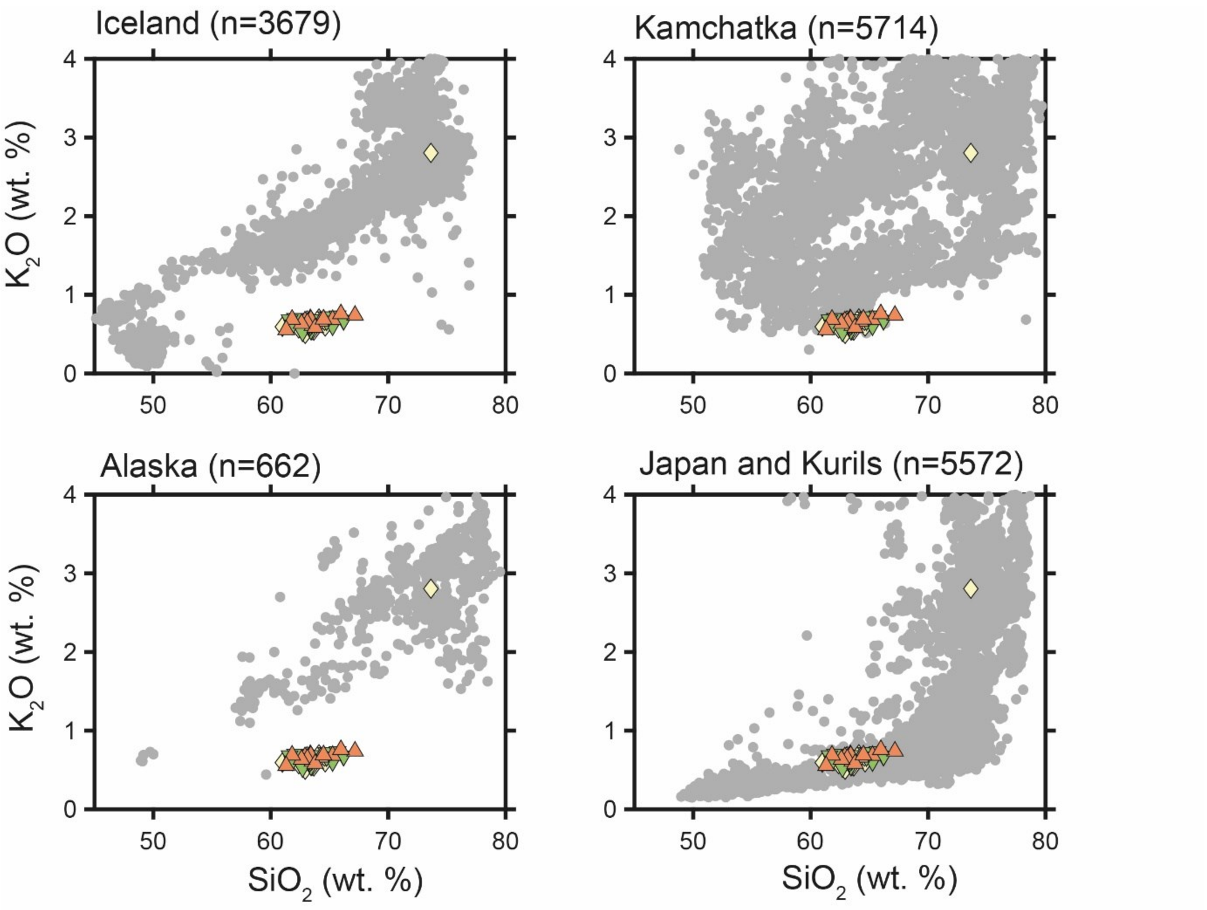

Figure S4: Ice core cryptotephra (coloured symbols) compared to regional tephra chemistry data sets (grey symbols). Iceland data are from Tephrabase [https://www.tephrabase.org/, (20)], Kamchatka data are from TephraKam (21), Alaska data are from the Alaska Volcano Observatory tephra database (22) while Japan and Kuril data are from lake and marine cores (23–28).

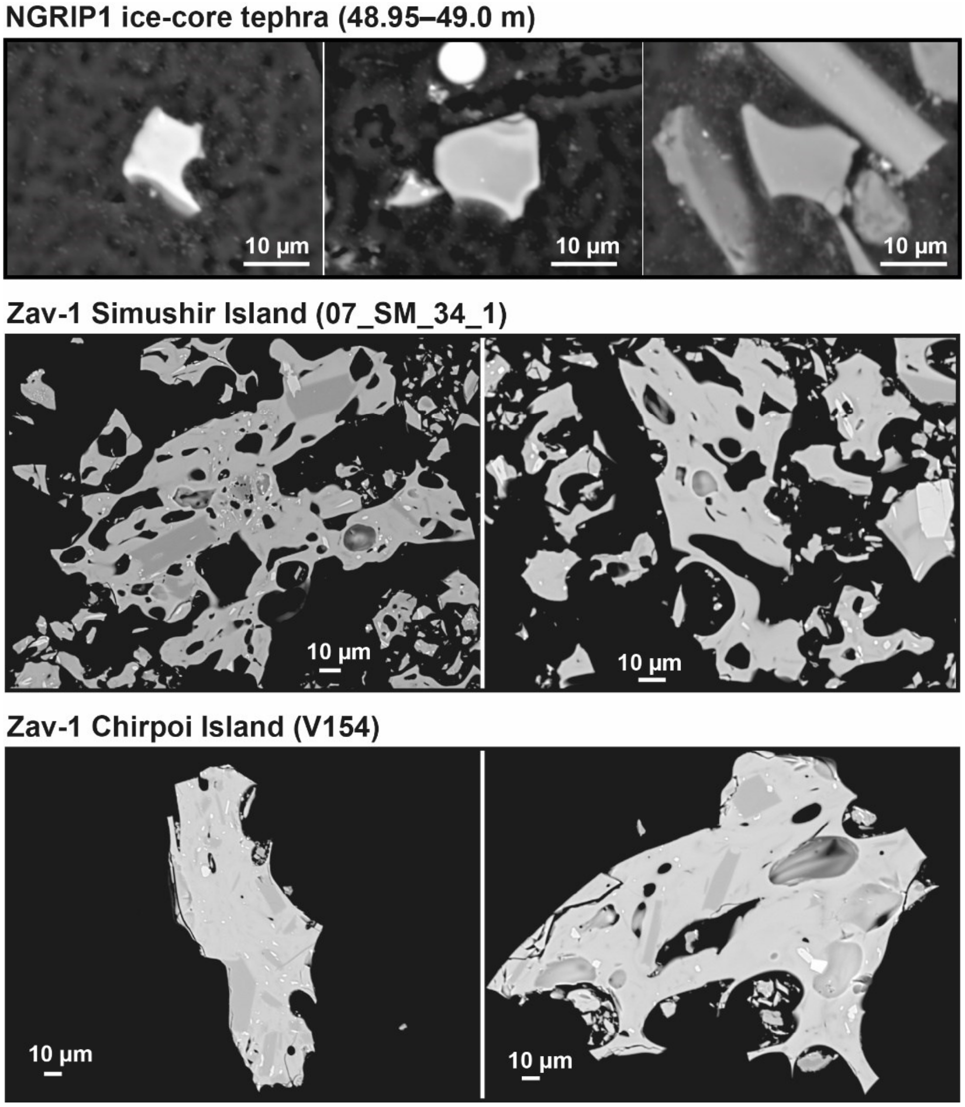

Figure S5: Images showing typical glass shards identified in this study. The images were acquired by scanning electron microscopy (SEM) using the backscattered electron detector (BED-C, compositional image). In the Zav-1 proximal samples from Simushir and Chirpoi Islands the bright and dark inclusions within the shards represent microlites of feldspar, pyroxene and Fe-Ti oxides.

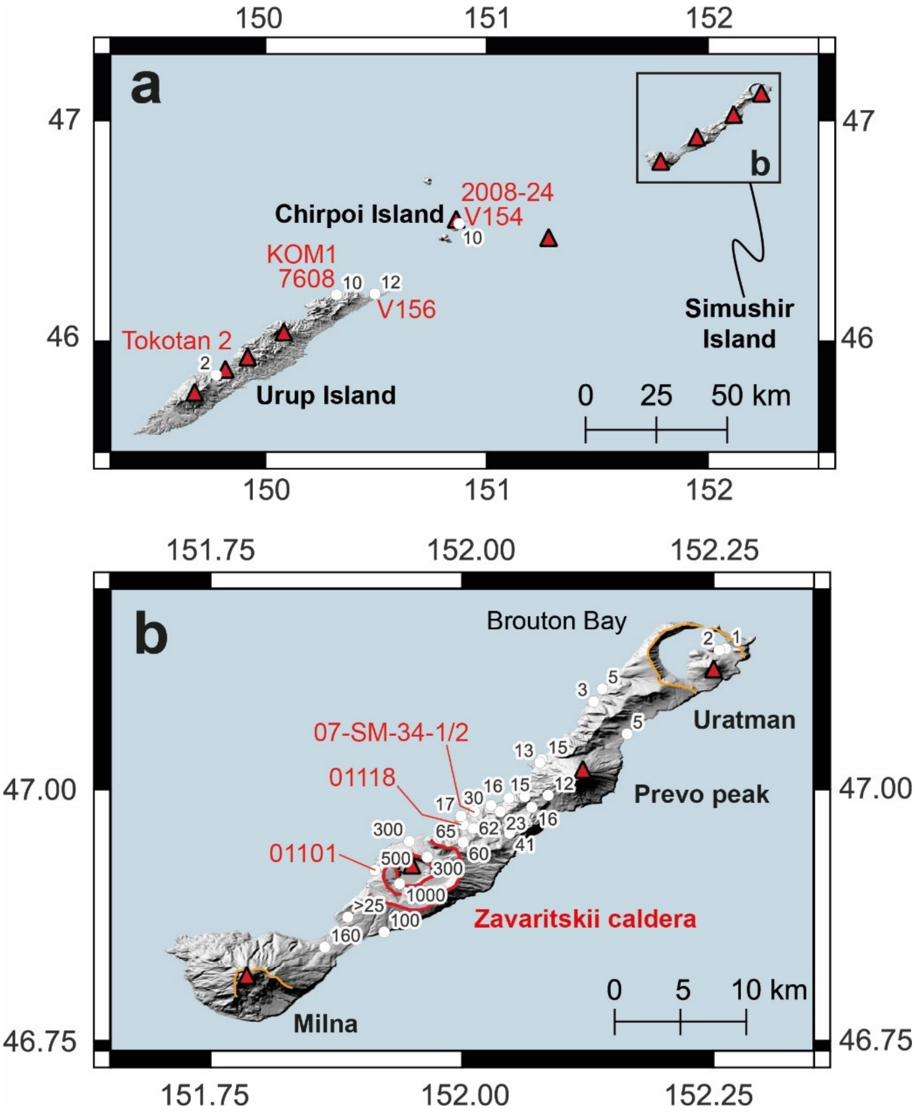

Figure S6: Thicknesses of Zav-1 tephra (in cm) on Simushir, Chirpoi and Urup island. EPMA data for Zav-1 tephra were collected for the sites labelled by red text (detailed in Dataset S3). Volcanoes are identified by red triangles and their calderas are outlined (in orange and red).

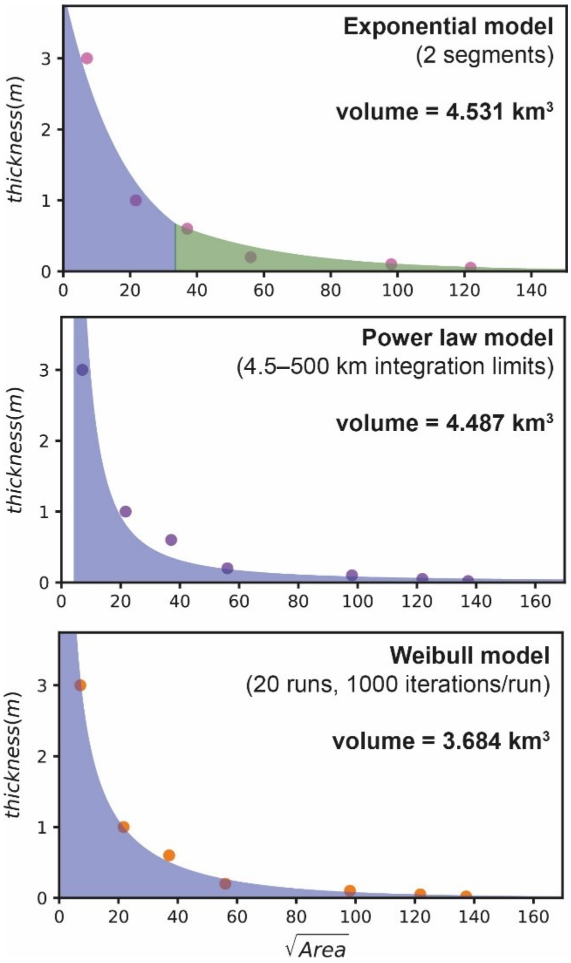

Figure S7: Volume estimates of the Zav-1 tephra fall deposits. These were calculated using AshCalc (29) and show the the three most commonly used models: the exponential model (30), the power law model (31, 32) and the Weibull model (33, 34).

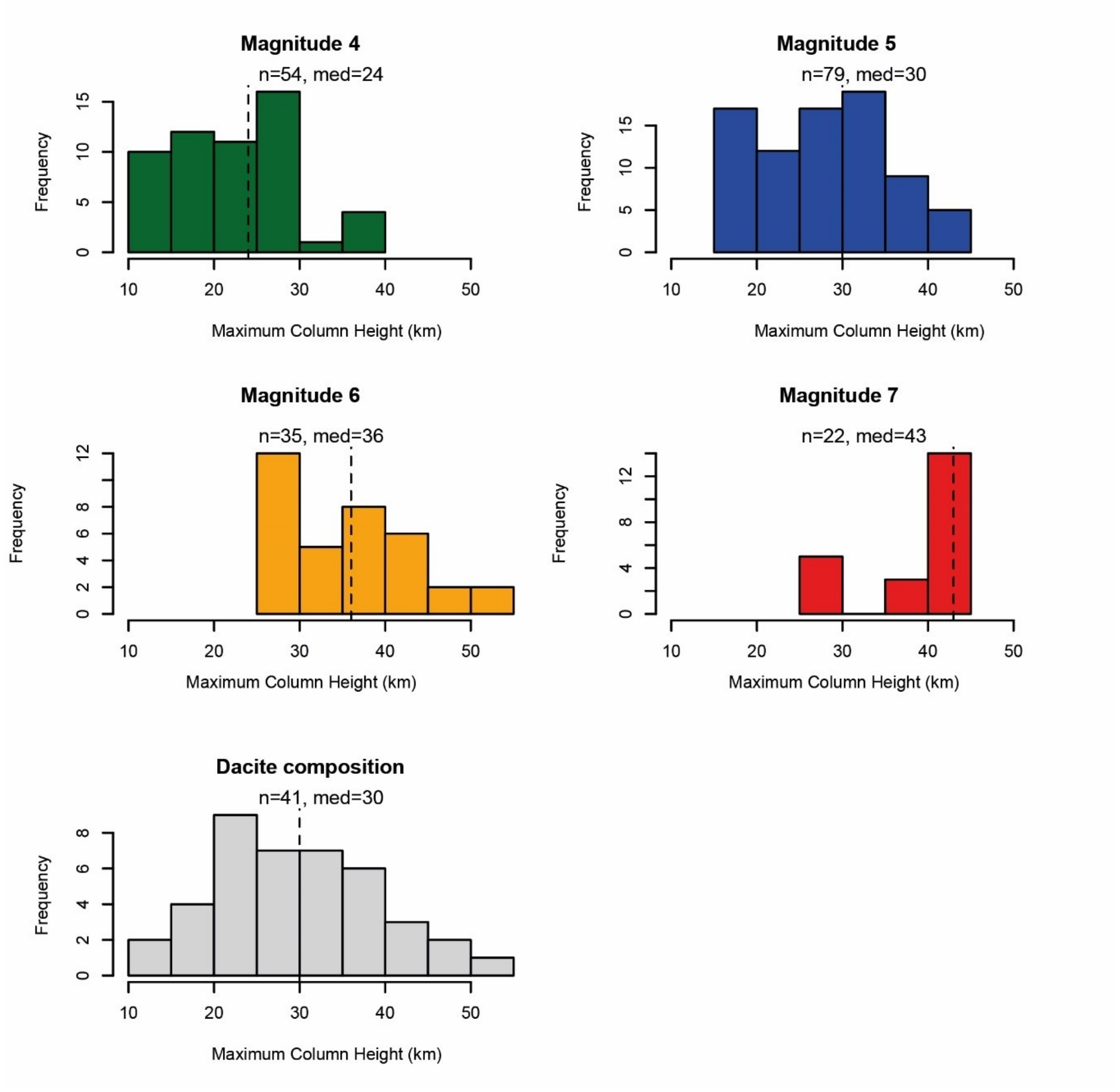

Figure S8: Maximum column height distributions for magnitude 4–7 eruptions and dacitic compositions on the LaMEVE (Large Magnitude Explosive Volcanic Eruptions) database (35). Note that LaMEVE contains ~2000 eruptions spanning the last 1.8 Ma with magnitude ≥4. In each plot the median (med) value is shown by the dotted vertical line. The 1831 CE eruption of Zavaritskii (Zav-1) is a magnitude 5–6 and so we use the median plume height range of 30–36 km for reconstructing stratospheric aerosol optical depth and radiative forcing for this event (detailed in Methods).

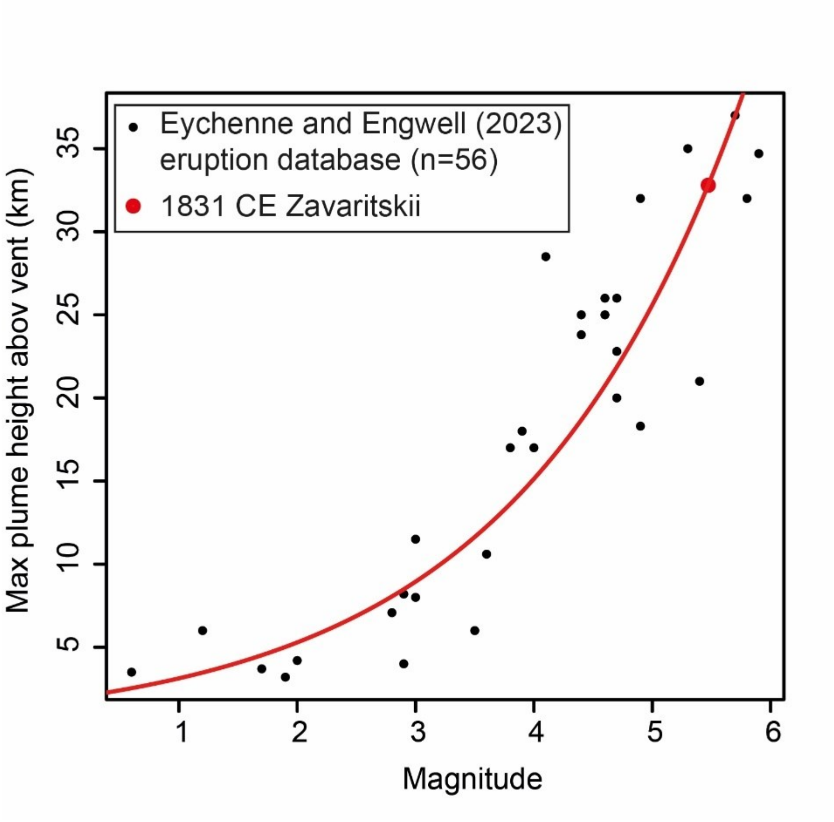

Figure S9: Maximum column height versus magnitude for a global data set of tephra fall deposits from Eychenne and Engwell (36). We fit the trend with an exponential function: $ln_{plume\ height\ above\ vent\ (km)} = 0.61457 + 0.52554\ \times \mathrm{magntiude}$. Using the magnitude estimated for the 1831 CE Zav-1 tephra (5.5, see main text) we calculate a plume height of 33 km above vent.

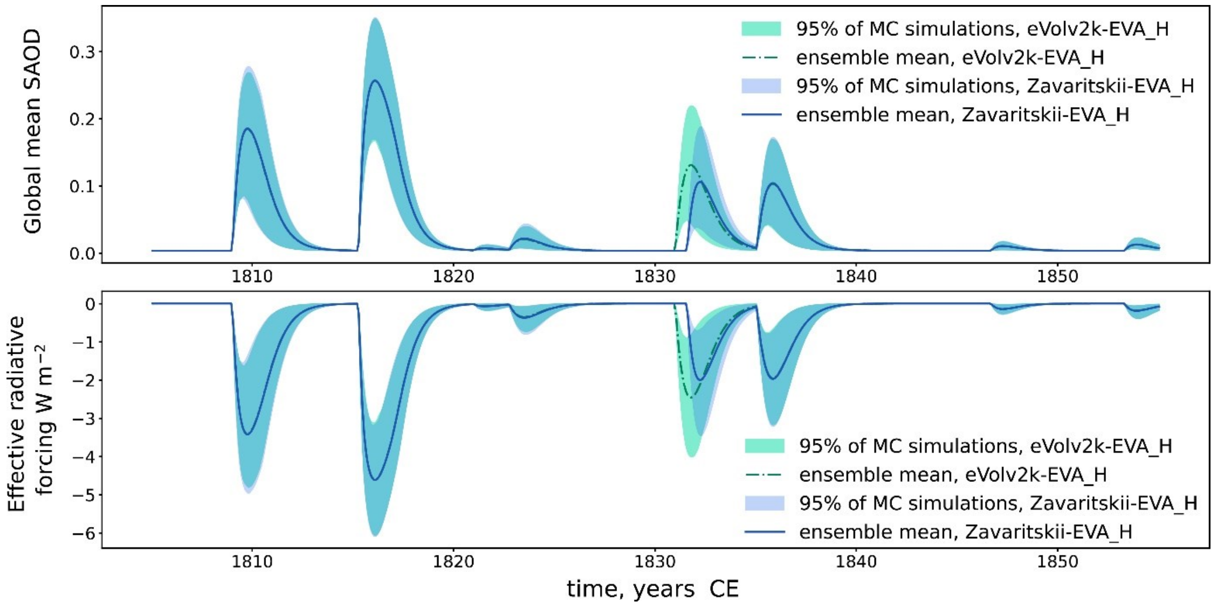

Figure S10: Global mean stratospheric aerosol optical depth (SAOD, 550 nm) and effective radiative forcing from the extended Easy Volcanic Aerosol Model [EVA_H, (37)]. The reconstruction uses volcanic stratospheric S constraints from an array of bipolar ice core records [i.e. eVolv2k, (19)]. Note that the Zavaritskii-EVA_H model runs includes updated parameters for the 1831 CE eruption of Zavaritskii (Zav-1, i.e. latitude = 46.9 °N, SO_2 height = 23 ± 12 km above sea level, stratospheric S mass = 12 ± 7 Tg and date = 01/08/1831).

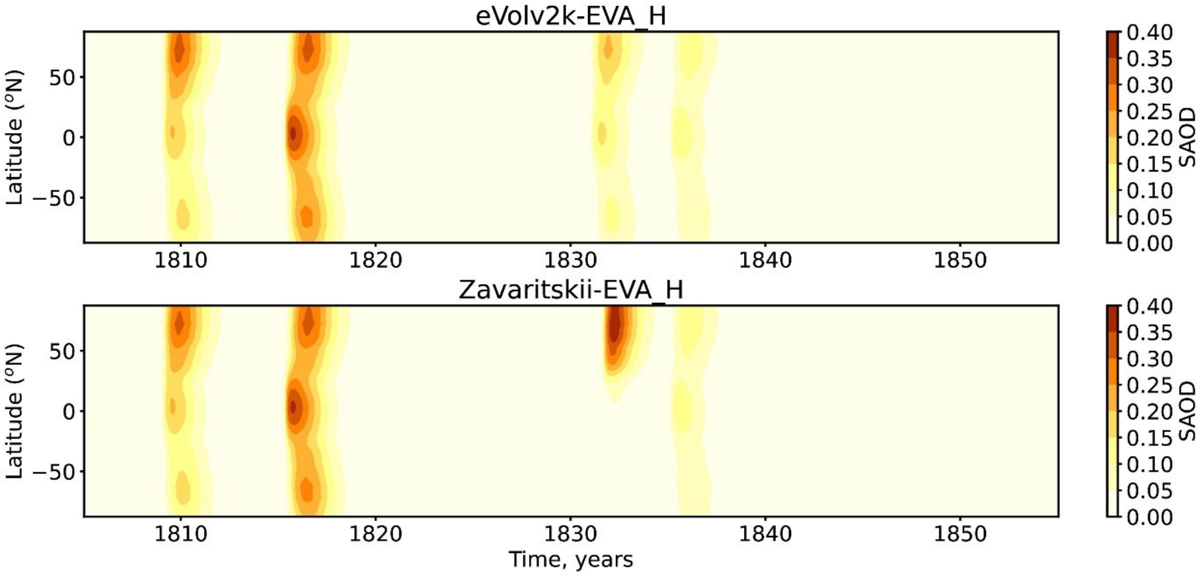

Figure S11: The time and latitude evolution of stratospheric aerosol optical depth (SAOD, 550 nm) for the different volcanic reconstructions.

Supplementary Table

Table S1: Radiocarbon dates constraining the age of Zav-1 (the youngest pumiceous deposits of Zavaritskii caldera). Radiocarbon dates were calibrated with IntCal20 (38) and OxCal v.4.4 (39). Those dates highlighted with an asterisk (*) indicate radiocarbon near or beyond the end of the calibration curve. Precise radiocarbon dating of materials from the 19th century is challenging because of the plateau in the radiocarbon calibration curve between 1700 and 1950 CE.

sample	latitude	longitude	collector/reference	material and sample context	conventional age (2σ) (^{14}C yr BP)	calibrated age (mean ± 1σ) (cal CE)	calibrated age range (2σ) (cal CE)
2153c	46.9844	152.0287	J. Bourgeois	Simushir Island, Charcoal below Zav-1 tephra	105 ± 25	1822 ± 76	1670–1950*
2153b	46.9844	152.0287	J. Bourgeois	Simushir Island, Charcoal below Zav-1 tephra	140 ± 25	1814 ± 82	1650–1950*
01118/A1	46.96	152.00	O. Dirksen	Simushir Island, Charcoal within Zav-1 tephra	260 ± 25	1642 ± 73	1496–1788
01118/A3	46.96	152.00	O. Dirksen	Simushir Island, Charcoal within Zav-1 tephra	290 ± 25	1579 ± 50	1479–1679
1/11307	46.9755	152.0021	Razzhigaeva et al. (2013) (2)	Simushir Island, Wood within syn-eruptive mudflow deposits	370 ± 80	1548 ± 83	1382–1714
2/11307	46.9755	152.0021	Razzhigaeva et al. (2013) (2)	Simushir Island, Wood within syn-eruptive mudflow deposits	380 ± 50	1535 ± 61	1413–1657
3/11307	46.9755	152.0021	Razzhigaeva et al. (2013) (2)	Simushir Island, Charcoal within syn-eruptive mudflow deposits	35 ± 50	1820 ± 77	1666–1950*
1/10007	46.9801	152.0135	Razzhigaeva et al. (2013) (2)	Simushir Island, Soil below Zav-1 tephra	660 ± 50	1336 ± 39	1258–1414
1/5607	46.8439	151.8652	Razzhigaeva et al. (2013) (2)	Simushir Island, Soil below Zav-1 tephra	600 ± 50	1354 ± 36	1282–1426
1a/1607	47.0539	152.1634	Razzhigaeva et al. (2013) (2)	Simushir Island, Peat below Zav-1 tephra	40 ± 90	1809 ± 84	1641–1950*
IKIP 0215, A40945	46.5402	150.8992	Fitzhugh et al. (2002) (4)	Chirpoi Island, Peschanaya Bay, House 31, hearth fill containing rusted gun fragments and muscovite imported for window panes by Russians in colonial period (1700s). Cultural deposits beneath 10 cm ash	162 ± 40	1800 ± 86	1628–1950*
KBP 0421, OS-59415	46.2118	150.3168	J. Bourgeois; Fitzhugh et al (2016) (5)	Urup Island, Charcoal below Zav-1 tephra	170 ± 30	1794 ± 86	1622–1950*

SI References

1. M. Nakagawa, Y. Ishizuka, T. Hasegawa, A. Baba, A. Kosugi, "Preliminary Report on Volcanological Research of KBP 2007-08 Cruise by Japanese Volcanology group" (2008).
2. N. G. Razzhigaeva, *et al.*, Role of climatic and volcanogenic factors in the formation of organogenic sediments and the development of landscapes on Simushir Island (Central Kurile Islands) in the middle-late Holocene. *Russ. J. Pacific Geol.* **7**, 199–211 (2013).
3. B. MacInnes, E. Kravchunovskaya, T. Pinegina, J. Bourgeois, Paleotsunamis from the central Kuril Islands segment of the Japan-Kuril-Kamchatka subduction zone. *Quat. Res. (United States)* **86**, 54–66 (2016).
4. B. Fitzhugh, V. O. Shubin, K. Tezuka, Y. Ishizuka, C. A. S. Mandryk, Archaeology in the Kuril Islands: Advances in the study of human paleobiogeography and Northwest Pacific prehistory. *Arctic Anthropol.* **39**, 69–94 (2002).
5. B. Fitzhugh, E. W. Gjesfjeld, W. A. Brown, M. J. Hudson, J. D. Shaw, Resilience and the population history of the Kuril Islands, Northwest Pacific: A study in complex human ecodynamics. *Quat. Int.* **419**, 165–193 (2016).
6. B. MacInnes, N. Slobodina, KBP Archaeological and Geological Profile Correlations. *tDAR id 376298* (2010).
7. N. G. Razjigaeva, L. A. Ganzey, K. A. Arslanov, N. F. Pshenichnikova, Coastal dunes of Urup Island (Kuril Islands, North-Western Pacific): palaeoclimatic and environmental archive. *Geosystems Transit. Zo.* **6**, 100–113 (2022).
8. R. Rohde, *et al.*, A new estimate of the average Earth surface land temperature spanning 1753 to 2011. Geoinfor Geostat: An Overview 1: 1. (2013).
9. M. Stoffel, *et al.*, Estimates of volcanic-induced cooling in the Northern Hemisphere over the past 1,500 years. *Nat. Geosci.* **8**, 784–788 (2015).
10. L. Schneider, *et al.*, Revising midlatitude summer temperatures back to A.D. 600 based on a wood density network. *Geophys. Res. Lett.* **42**, 4556–4562 (2015).
11. R. Wilson, *et al.*, Last millennium northern hemisphere summer temperatures from tree rings: Part I: The long term context. *Quat. Sci. Rev.* **134**, 1–18 (2016).
12. K. J. Anchukaitis, *et al.*, Last millennium Northern Hemisphere summer temperatures from tree rings: Part II, spatially resolved reconstructions. *Quat. Sci. Rev.* **163**, 1–22 (2017).
13. A. Hernández, *et al.*, A 2,000-year Bayesian NAO reconstruction from the Iberian Peninsula. *Sci. Rep.* **10**, 14961 (2020).
14. M. E. Mann, *et al.*, Global Signatures and Dynamical Origins of the Little Ice Age and Medieval Climate Anomaly. *Science (80-.).* **326**, 1256–1260 (2009).
15. S. T. Gray, L. J. Graumlich, J. L. Betancourt, G. T. Pederson, A tree-ring based reconstruction of the Atlantic Multidecadal Oscillation since 1567 A.D. *Geophys. Res. Lett.* **31** (2004).
16. K. M. Keegan, M. R. Albert, J. R. McConnell, I. Baker, Climate change and forest fires synergistically drive widespread melt events of the Greenland Ice Sheet. *Proc. Natl. Acad. Sci. U. S. A.* **111**, 7964–7967 (2014).
17. J. R. McConnell, "D4 continuous ice core chemistry measurements below pore closeoff." (2016).
18. C. T. Plummer, *et al.*, An independently dated 2000-yr volcanic record from Law Dome, East Antarctica, including a new perspective on the dating of the 1450s CE eruption of Kuwae, Vanuatu. *Clim. Past* **8**, 1929–1940 (2012).
19. M. Toohey, M. Sigl, Volcanic stratospheric sulfur injections and aerosol optical depth from 500 BCE to 1900 CE. *Earth Syst. Sci. Data* **9**, 809–831 (2017).
20. A. J. Newton, A. J. Dugmore, B. M. Gittings, Tephrabase: tephrochronology and the development of a centralised European database. *J. Quat. Sci.* **22**, 737–743 (2007).
21. M. V. Portnyagin, *et al.*, TephraKam: Geochemical database of glass compositions in tephra and welded tuffs from the Kamchatka volcanic arc (northwestern Pacific). *Earth Syst. Sci. Data* **12**, 469–486 (2020).
22. K. L. Wallace, Alaska Tephra Data. *U.S. Geol. Surv. data release* (2018). Available at: https://doi.org/10.5066/P9PFQGVC.
23. V. C. Smith, *et al.*, Identification and correlation of visible tephras in the Lake Suigetsu SG06 sedimentary archive, Japan: Chronostratigraphic markers for synchronising of east

Asian/west Pacific palaeoclimatic records across the last 150 ka. *Quat. Sci. Rev.* **67**, 121–137 (2013).
24. N. G. Razzhigaeva, A. Matsumoto, M. Nakagawa, Age, source, and distribution of Holocene tephra in the southern Kurile Islands: Evaluation of Holocene eruptive activities in the southern Kurile arc. *Quat. Int.* **397**, 63–78 (2016).
25. X.-Y. Chen, *et al.*, Clarifying the distal to proximal tephrochronology of the Millennium (B–Tm) eruption, Changbaishan Volcano, northeast China. *Quat. Geochronol.* **33**, 61–75 (2016).
26. X.-Y. Chen, *et al.*, Developing a Holocene tephrostratigraphy for northern Japan using the sedimentary record from Lake Kushu, Rebun Island. *Quat. Sci. Rev.* **215**, 272–292 (2019).
27. J. C. Schindlbeck, *et al.*, One Million Years tephra record at IODP Sites U1436 and U1437: Insights into explosive volcanism from the Japan and Izu arcs. *Isl. Arc* **27**, 1–16 (2018).
28. P. G. Albert, *et al.*, Geochemical characterisation of the Late Quaternary widespread Japanese tephrostratigraphic markers and correlations to the Lake Suigetsu sedimentary archive (SG06 core). *Quat. Geochronol.* **52**, 103–131 (2019).
29. M. L. Daggitt, T. A. Mather, D. M. Pyle, S. Page, AshCalc-a new tool for the comparison of the exponential, power-law and Weibull models of tephra deposition. *J. Appl. Volcanol.* **3**, 1–8 (2014).
30. D. M. Pyle, The thickness, volume and grainsize of tephra fall deposits. *Bull. Volcanol.* **51**, 1–15 (1989).
31. C. Bonadonna, G. G. J. Ernst, R. S. J. Sparks, Thickness variations and volume estimates of tephra fall deposits: the importance of particle Reynolds number. *J. Volcanol. Geotherm. Res.* **81**, 173–187 (1998).
32. C. Bonadonna, B. F. Houghton, Total grain-size distribution and volume of tephra-fall deposits. *Bull. Volcanol.* **67**, 441–456 (2005).
33. C. Bonadonna, A. Costa, Estimating the volume of tephra deposits: A new simple strategy. *Geology* **40**, 415–418 (2012).
34. C. Bonadonna, A. Costa, Plume height, volume, and classification of explosive volcanic eruptions based on the Weibull function. *Bull. Volcanol.* **75**, 742 (2013).
35. H. S. Crosweller, *et al.*, Global database on large magnitude explosive volcanic eruptions (LaMEVE). *J. Appl. Volcanol.* **1**, 1–13 (2012).
36. J. Eychenne, S. L. Engwell, The grainsize of volcanic fall deposits: Spatial trends and physical controls. *Bull. Geol. Soc. Am.* **135**, 1844–1858 (2023).
37. T. J. Aubry, M. Toohey, L. Marshall, A. Schmidt, A. M. Jellinek, A New Volcanic Stratospheric Sulfate Aerosol Forcing Emulator (EVA_H): Comparison With Interactive Stratospheric Aerosol Models. *J. Geophys. Res. Atmos.* **125** (2020).
38. P. J. Reimer, *et al.*, The IntCal20 Northern Hemisphere radiocarbon age calibration curve (0–55 cal kBP). *Radiocarbon* **62**, 725–757 (2020).
39. C. Bronk Ramsey, Bayesian Analysis of Radiocarbon Dates. *Radiocarbon* **51**, 337–360 (2009).
